# Functional connectivity signatures of NMDAR dysfunction in schizophrenia—integrating findings from imaging genetics and pharmaco-fMRI

**DOI:** 10.1038/s41398-023-02344-2

**Published:** 2023-02-16

**Authors:** Arnim J. Gaebler, Nilüfer Fakour, Felix Stöhr, Jana Zweerings, Arezoo Taebi, Mariia Suslova, Juergen Dukart, Joerg F. Hipp, Bhim M. Adhikari, Peter Kochunov, Suresh D. Muthukumaraswamy, Anna Forsyth, Thomas Eggermann, Florian Kraft, Ingo Kurth, Michael Paulzen, Gerhard Gründer, Frank Schneider, Klaus Mathiak

**Affiliations:** 1 Department of Psychiatry, Psychotherapy and Psychosomatics, Faculty of Medicine, RWTH Aachen, Aachen, Germany; 2 JARA - Translational Brain Medicine, Aachen, Germany; 3 Department of Physiology, Faculty of Medicine, RWTH Aachen, Aachen, Germany; 4 F. Hoffmann-La Roche, Pharma Research Early Development, Roche Innovation Centre Basel, Basel, Switzerland; 5 Institute of Neuroscience and Medicine, Brain & Behaviour (INM-7), Research Centre Jülich, Jülich, Germany; 6 Institute of Systems Neuroscience, Medical Faculty, Heinrich Heine University Düsseldorf, Düsseldorf, Germany; 7 Maryland Psychiatric Research Center, Department of Psychiatry, University of Maryland School of Medicine, Baltimore, MD USA; 8 School of Pharmacy, Faculty of Medical and Health Sciences, The University of Auckland, Auckland, New Zealand; 9 Institute for Human Genetics and Genomic Medicine, Faculty of Medicine, RWTH Aachen, Aachen, Germany; 10 Alexianer Hospital, Aachen, Germany; 11 Central Institute of Mental Health, Department of Molecular Neuroimaging, Medical Faculty Mannheim, University of Heidelberg, Mannheim, Germany; 12 University Hospital Düsseldorf, Heinrich-Heine-University, Düsseldorf, Germany

**Keywords:** Schizophrenia, Molecular neuroscience

## Abstract

Both, pharmacological and genome-wide association studies suggest N-methyl-D-aspartate receptor (NMDAR) dysfunction and excitatory/inhibitory (E/I)-imbalance as a major pathophysiological mechanism of schizophrenia. The identification of shared fMRI brain signatures of genetically and pharmacologically induced NMDAR dysfunction may help to define biomarkers for patient stratification. NMDAR-related genetic and pharmacological effects on functional connectivity were investigated by integrating three different datasets: (A) resting state fMRI data from 146 patients with schizophrenia genotyped for the disease-associated genetic variant rs7191183 of GRIN2A (encoding the NMDAR 2 A subunit) as well as 142 healthy controls. (B) Pharmacological effects of the NMDAR antagonist ketamine and the GABA-A receptor agonist midazolam were obtained from a double-blind, crossover pharmaco-fMRI study in 28 healthy participants. (C) Regional gene expression profiles were estimated using a postmortem whole-brain microarray dataset from six healthy donors. A strong resemblance was observed between the effect of the genetic variant in schizophrenia and the ketamine versus midazolam contrast of connectivity suggestive for an associated E/I-imbalance. This similarity became more pronounced for regions with high density of NMDARs, glutamatergic neurons, and parvalbumin-positive interneurons. From a functional perspective, increased connectivity emerged between striato-pallido-thalamic regions and cortical regions of the auditory-sensory-motor network, while decreased connectivity was observed between auditory (superior temporal gyrus) and visual processing regions (lateral occipital cortex, fusiform gyrus, cuneus). Importantly, these imaging phenotypes were associated with the genetic variant, the differential effect of ketamine versus midazolam and schizophrenia (as compared to healthy controls). Moreover, the genetic variant was associated with language-related negative symptomatology which correlated with disturbed connectivity between the left posterior superior temporal gyrus and the superior lateral occipital cortex. Shared genetic and pharmacological functional connectivity profiles were suggestive of E/I-imbalance and associated with schizophrenia. The identified brain signatures may help to stratify patients with a common molecular disease pathway providing a basis for personalized psychiatry.

## Introduction

**Fig. 1:**
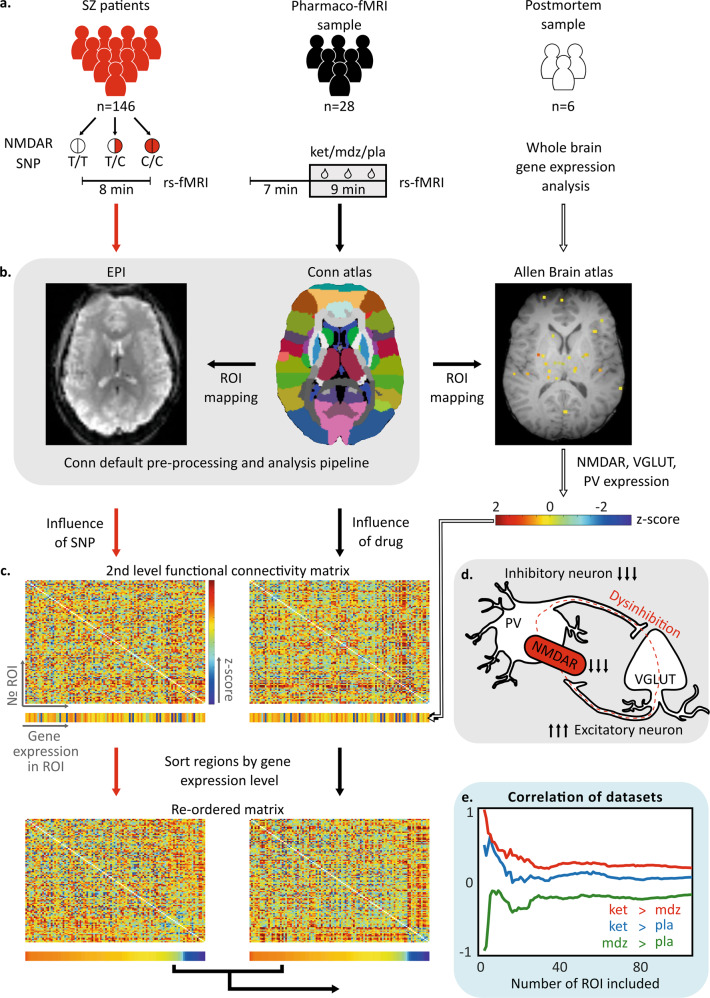
Study synopsis. **a** In total, three different datasets were analyzed: (1) resting state-fMRI (rs-fMRI) data from 146 patients with schizophrenia genotyped for the disease-associated single nucleotide polymorphism (SNP) of the NMDAR; (2) resting state-fMRI data from 28 healthy subjects receiving ketamine (ket), midazolam (mdz) and placebo (pla) within a three-way cross-over pharmaco-fMRI study; and (3) postmortem whole-brain microarray gene expression data from six healthy subjects. T/T, T/C, and C/C refer to wild-type, heterozygous, and homozygous genotype, respectively. **b** All fMRI data were subjected to the default pre-processing and analysis pipeline of the conn toolbox, including a parcellation of the spatially normalized brains according to its standard brain atlas. Tissue samples of the spatially normalized postmortem brains were also mapped onto the Harvard Oxford Atlas (here referred to as Conn atlas) in order to link the functional connectivity estimates of the respective regions of interest (ROI) to their gene expression profiles. EPI echoplanar imaging. **c** According to the parcellation of the Harvard Oxford Atlas, a 106 × 106 s level matrix was generated for each of the two fMRI datasets, assessing the impact of the number of SNP alleles or the pharmacological contrast, respectively, on functional connectivity within the framework of a general linear model. Regions were subsequently sorted by their average expression of three target genes including the constitutive subunit of the NMDAR (GRIN1), the glutamatergic marker gene SLC17A7 encoding the vesicular glutamate transporter VGLUT1, and parvalbumin (PV). **d** Schema of the pathophysiological model of the E/I-imbalance in schizophrenia which served as the rationale for the selection of the three target genes: Reduced NMDAR function on parvalbumin-positive GABAergic interneurons leads to a disinhibition of glutamatergic (i.e. VGLUT-positive) pyramidal cells. **e** Correlation between the functional connectivity changes associated with the SNP and the three different pharmacological contrasts was assessed for varying numbers of the genetically ranked regions (for a more detailed overview of the results see Fig. 2).

The pathophysiology of schizophrenia is still enigmatic. One of the most compelling hypotheses suggests a dysfunction of N-methyl-D-aspartate (NMDA) receptors as a major disease mechanism [1–3]. Historically, the larger body of evidence has been indirect, relying predominantly on pharmacological models applying sub-anesthetic doses of NMDAR antagonists such as ketamine and phencyclidine which induce a clinical phenotype mimicking various symptoms of schizophrenia in healthy subjects [4]. At the microcircuit level, these models suggest that NMDAR hypofunction in schizophrenia is considered to mainly affect parvalbumin-positive GABAergic interneurons leading to a disinhibition of glutamatergic pyramidal cells and consequently an imbalance of neural excitation and inhibition (E/I-imbalance, see Fig. 1d) [2, 5]. More direct evidence for the NMDAR hypothesis of schizophrenia has been recently provided by large genetic studies which revealed that both common and rare genetic variants associated with schizophrenia converge to genes related to NMDAR functioning [6–9]. Among these genes, GRIN2A, which encodes the NMDAR 2A (NR2A or GluN2A) subunit, emerged as one of the most highly prioritized genes related to the pathophysiology of schizophrenia [6, 7, 10]. Besides the rarely occurring exonic variants of GRIN2A [7], genome-wide association studies (GWAS) consistently identified a set of common intronic single nucleotide polymorphisms (SNP) of this gene to be associated with the disease [6, 8, 9]. These SNPs are in a tight linkage disequilibrium [6, 8] and the respective disease-associated variants are quite common in the general population exhibiting allele frequencies around 30% (see dbSNP [11]). Previous research suggests that the respective variants promote DNA methylation at GRIN2A-related CpG-sites [10] and thus lead to reduced GRIN2A expression. Indeed, Quantitative trait loci (QTL) databases provide further evidence for GRIN2A hypermethylation and reduced transcript expression associated with the GRIN2A variant investigated in this study (see Supplementary Results R1). Despite this first evidence on the level of gene expression, the neurophysiological or behavioral consequences of these common intronic variants are still unknown, but may be partly estimated from the effects of rarely occurring exonic loss-of-function variants. Besides schizophrenia, these exonic variants are typically associated with prominent language-related symptoms and epilepsy [12, 13] with the latter manifestation pointing to severe E/I-imbalance. Neural hyperexcitability in GRIN2A loss of function variants has been related to hypofunction of Parvalbumin-positive interneurons as NR2A-containing NMDA receptors are highly expressed and pivotal in the maintenance of this subgroup of interneurons [14, 15]. Integrating these different streams of evidence, we assumed that the common schizophrenia-associated intronic variants of GRIN2A lead to reduced expression of the NR2A subunit, which should particularly affect Parvalbumin-positive interneurons and thus cause a disinhibition of glutamatergic pyramidal cells (Fig. 1d) as suggested both by pharmacological models of schizophrenia and the phenotype of exonic GRIN2A loss of function variants.

The identification of shared pharmacologically and genetically induced neurophysiological signatures as well as behavioral phenotypes related to NMDAR dysfunction and E/I-imbalance may help to detect subgroups of patients who may benefit from tailored therapy. To meet this need, in the present study, we integrated different datasets combining imaging genetics, pharmaco-fMRI, and postmortem gene expression data. The neural correlates of a schizophrenia-related GRIN2A- variant were investigated by assessing resting state functional connectivity profiles in a sample of 146 patients with schizophrenia who were genotyped for this SNP. Corresponding pharmacological effects were addressed using a dataset from a previously published double blind crossover pharmaco-fMRI study [16] applying the NMDAR antagonist ketamine, the GABA-A receptor agonist midazolam and placebo. Based on our E/I-imbalance model, we hypothesized that the effect of the genetic variant would show greatest resemblance to the differential effect of (sub-anesthetic) ketamine versus midazolam as both drugs exhibit opposite effects on neural excitation/inhibition. Moreover, we assumed that this resemblance should be more pronounced for regions exhibiting a high density of NMDARs, parvalbumin-positive interneurons and glutamatergic neurons. Hence, we included a publicly available postmortem whole-brain gene expression dataset in our analysis in order to match regions exhibiting connectivity changes to their estimated expression level of the target genes [17]. In the next step, we assessed the impact of the genetic variant on the functional connectivity profile of different brain regions and networks which were either selected based on their estimated gene expression profile, findings from the literature on pharmacological models of NMDAR dysfunction or in a data-driven manner. Connections which were significantly affected by the genetic variant were subsequently tested in the pharmaco-fMRI dataset for their modulation by ketamine versus midazolam and in a combined schizophrenia-control dataset for their association with schizophrenia. Based on the literature, one of the most consistent resting state-fMRI findings which is shared both by patients with schizophrenia and ketamine models is a hyper-connectivity between the thalamus and the auditory-sensory-motor (ASM) network [18–20]. Recently, it was demonstrated in patients with schizophrenia that this pattern of hyper-connectivity is not restricted to the thalamus, but also involves the basal ganglia, particularly the caudate and the pallidum [21]. To the best of our knowledge, this extension of hyper-connectivity to the basal ganglia has not been addressed by ketamine studies yet and no information on the effect of the genetic variant is available. Therefore, we specifically assessed functional connectivity between striato-pallido-thalamic regions and cortical regions of the ASM network for the different datasets and effects of interest.

Furthermore, we investigated the clinical symptomatology associated with the GRIN2A variant, applying the Bern psychopathology scale [22, 23]. This clinical rating scale assigns symptoms to three different categories corresponding to well-defined brain systems, i.e., language, affective, and motor symptoms, and differentiates between over- and hypoactive manifestations of each item. Since the NMDAR hypothesis of schizophrenia is especially used for the explanation of negative symptoms [24], we hypothesized that the genetic variant is associated with negative symptomatology, i.e., hypoactive manifestations of the respective items. Finally, we tried to identify functional connectivity changes potentially mediating the clinical symptoms associated with the genetic variant.

## Materials and methods

We collected resting state-fMRI, genetic and clinical data from 146 in- and out-patients suffering from schizophrenia. To link the obtained resting state-fMRI connectivity findings of the genetic variant to the pathophysiology of schizophrenia, resting state fMRI data from 142 healthy control subjects were compared with the whole sample of patients suffering from schizophrenia. The healthy control subjects participated in different study arms of the same research network (APIC), but no genetic information was available. For the comparison of genetic and pharmacological brain signatures of NMDAR dysfunction, we also analyzed a pharmaco-fMRI dataset comprising resting state—fMRI data from 28 healthy male subjects who participated in a single-blind, placebo-controlled study with a three-way cross-over design applying ketamine, midazolam and placebo [16, 25, 26]. For all studies, written informed consent was obtained from all participants, following a complete description of the study. All studies were approved by the respective local authority (For more information on the characteristics of the study samples and respective study designs see Supplementary Methods M1 as well as Supplementary Tables 1 and 2, for information on genotyping see Supplementary Methods M2).

### Clinical assessment

We assessed the patients’ symptomatology, applying the Bern Psychopathology Scale, which assigns symptoms to three different domains related to well-defined brain circuits (language, affective, and motor symptoms) [22, 23]. Each domain is further divided in different subcategories and each item is rated on a bipolar axis (including a negative/hypoactive [−1], a positive/hyperactive [+1], and an average manifestation [0]). In order to assess the realtionship between the genetic variant and the global symptom load within each domain, we also applied a multivariate general linear model to each domain using the sum scores of the respective symptom subcategories as the dependent variables and the number of disease-associated C-alleles as the independent variable (For more details see the Supplementary Methods M3).

### Functional connectivity analysis

All fMRI datasets were subjected to the default pre-processing pipeline as implemented in the MATLAB conn toolbox. (For an overview of the MRI sequence parameters and the preprocessing steps see the supplementary material (Supplementary Methods M5 & M6). In order to link the functional connectivity estimates of the respective regions of interest (ROI) to their cellular and molecular architecture, we estimated their gene expression profiles from a postmortem whole-brain microarray dataset (Allen Human Brain atlas) [17]. Thereto, both the tissue samples of the postmortem brains and the different fMRI datasets were spatially normalized and mapped onto the Harvard Oxford Atlas (see above).

We conducted both ROI-to-ROI and seed-to-voxel functional connectivity analyses. In the first level analyses, Pearson correlation coefficients were calculated between the fMRI time series of the respective ROI-ROI or ROI-voxel pairs. For the pharmaco-fMRI dataset, correlation coefficients were calculated for each of the six pharmacological conditions separately (ketamine, midazolam as well as placebo pre and post infusion, respectively). In the second level analyses, the individual Fisher-z-transformed correlation coefficients served as dependent variables of a general linear model. To study the impact of the SNP on functional connectivity in the schizophrenia dataset, the number of disease-associated C-alleles (i.e. 0 for wildtype, 1 for heterozygous, and 2 for homozygous) served as the predictor of interest and the respective regression coefficients entered a one sample t-test when hypothesis testing was necessary. We chose this approach as we assumed an additive genetic model as commonly employed in Expression QTL (eQTL) analyses [27, 28] and suggested by the findings obtained from our own eQTL database query (see Supplementary Results R1). In an additional exploratory analysis, we treated the genotype as a categorical variable differentiating between the absence and the presence of the minor allele (see Supplementary Results R3). Since the pharmaco-fMRI dataset comprised only healthy male subjects, for a better comparability, we corrected for gender and medication effects in all (fMRI and behavioral) analyses conducted on the schizophrenia dataset by including gender and haloperidol equivalent doses [29] as predictors of no interest in the second level general linear model. These predictors were also included for comparisons between the whole schizophrenia dataset and the healthy control dataset.

In the pharmaco-fMRI dataset, the different pharmacological effects on functional connectivity were assessed by the contrasts

1. ketamine > placebo, i.e. (ketamine post – ketamine pre) – (placebo post – placebo pre),
2. midazolam > placebo, i.e. (midazolam post – midazolam pre) – (placebo post – placebo pre)
3. ketamine > midazolam, i.e. (ketamin post – ketamine pre) - (midazolam post – midazolam pre).

For hypothesis testing, paired t-tests were employed.

To assess the similarity between the effect of the genetic variant and each of the three pharmacological effects on functional connectivity, we conducted a correlation analysis. Since we hypothesized that the correlation should depend on the expression of NMDARs and the density of Parvalbumin-positive interneurons as well as glutamatergic neurons, we ranked the regions of the Harvard Oxford Atlas according to their averaged expression (i.e. averaged z-values) of the three marker genes GRIN1 (encoding the constitutive NMDAR 1 subunit), Parvalbumin and SLC17A7 (encoding the ‘vesicular glutamate transporter 1’ - VGLUT1, the main marker indicating the glutamatergic identity of a neuron), as revealed by the analysis of the Allen Brain dataset (see Supplementary Methods M4 for a description of the analysis of gene expression data and Supplementary Table 3 for the overview of the regions in their ranked order). Note that we used the marker gene GRIN1 instead of GRIN2A as GRIN1 encodes the constitutive NMDAR 1 subunit. Accordingly, the impact of genetic variants of GRIN2A are functionally only relevant if GRIN1 is co-expressed. Moreover, expression of GRIN2A shows a stronger age-dependence [30, 31] and may consequently be less reliably estimated. Nevertheless, we also conducted exploratory analyses using GRIN2A instead of GRIN1 (see Supplementary Results R2, Supplementary Figs. 2 and 4)

Subsequently, we re-ordered the matrices of the averaged connectivity changes according to the ranks of the regions and repeatedly assessed the correlation, starting from the full 106 × 106 matrix of connectivity changes and iteratively removing the region with the lowest expression of the target genes until only the three highest expressing regions remained. Correlation was estimated by calculating Pearson correlation coefficients for the lower triangles of the respective symmetric connectivity submatrices remaining at each iteration. Due to the inherent interdependence of the whole-brain functional connectivity estimates, the significance of the correlation curves was assessed by comparing their area under the curve with a null distribution which was generated by 10,000 random permutations of genotype (i.e. in each iteration, genotype labels were randomly re-assigned to the patients and the second level analysis and all subsequent steps were re-done based on the permuted genotype, see Fig. 1 for an illustration of the methodological procedure).

Since the contrast ketamine > midazolam showed the highest similarity with the SNP effect in our functional connectivity analysis and—as shown before [16]—also exhibited the highest similarity with schizophrenia-associated connectivity alterations in general, we restricted the subsequent connectivity analyses of the pharmaco-fMRI dataset to this contrast.

In order to further explore the topography of the SNP-associated connectivity changes, we first conducted a seed to voxel analysis using the left cuneus as the seed, as it exhibited the highest average expression of our genes of interest in the postmortem dataset. The two strongest connectivity changes of this seed were subsequently re-assessed in the pharmaco-fMRI dataset for the contrast ketamine > midazolam as well as in the combined schizophrenia/control sample for the contrast schizophrenia > healthy controls using ROI-to-ROI analyses.

Connectivity between striato-pallido-thalamic regions and cortical regions of the auditory-sensory-motor (ASM) network was assessed, applying a multivariate ROI to ROI analysis which addressed the overall connectivity between the two sets of regions (functional network connectivity analysis of the Conn toolbox [32], for further details see supplementary methods M7 and Supplementary Table 5) for each contrast of interest.

To identify further NMDAR-related imaging phenotypes, we applied another functional network connectivity analysis studying the effect of the GRIN2A variant on a whole-brain level and in a hypothesis-free manner. This time, networks of ROIs were defined using a data-driven hierarchical clustering procedure. Subsequently, for each network, the within network connectivity and for each pair of networks, the between network connectivity were assessed, respectively. FDR-correction was applied to control for multiple testing on the network level (p-FDR < 0.05). Again, the two strongest connectivity changes related to the genetic variant were subsequently re-assessed for the other datasets/contrasts of interest, using ROI-to-ROI analyses (for more details see supplementary methods M8).

Finally, since it turned out that the GRIN2A-variant was associated with language-related negative symptomatology, we also aimed at identifying the underlying connectivity changes potentially mediating this effect. Since we hypothesized that the respective connections involved brain regions related to language function, we conducted a seed to voxel functional connectivity analysis using the left posterior superior temporal gyrus (pSTG, corresponding to Wernicke’s area) and the left inferior frontal gyrus (pars triangularis, corresponding to Broca’s area) of the Harvard Oxford Atlas as two separate seed regions. We first identified connections which were negatively affected by the genetic variant, applying a general linear model with the number of C-alleles constituting the predictor of interest Those connections were subsequently tested for their association with qualitative language symptoms (which showed strongest association with the genetic variant), applying a partial correlation analysis. Connections that exhibited a significant relationship both with the genetic variant and language symptomatology were further assessed for the two other datasets/contrasts of interest

For all seed to voxel analyses presented in this paper, we corrected for multiple testing by applying Gaussian Random Field theory with a combination of a height threshold of *p* < 0.001 at the voxel level and a FDR-corrected *p* < 0.05 cluster-size threshold, adhering to the standard implementation in the Conn toolbox. For the verification of specific connectivity changes associated with the genetic variant in the pharmaco-fMRI dataset or the combined schizophrenia/control dataset, one-tailed p-values were applied which were FDR-corrected. For all statistical tests, the respective underlying assumptions were controlled. For group comparisons, homogeneity of variance was confirmed using Levene’s test.

Statistical analyses and visualization of data were performed in Matlab 2020a (The MathWorks, Inc., Natick, USA), the Matlab conn toolbox, SPSS 27 (IBM, Armonk, USA), RStudio (RStudio Team (2021). RStudio: Integrated Development Environment for R. RStudio, PBC, Boston, MA URL http://www.rstudio.com/) and GraphPad Prism 9 (GraphPad Software, San Diego, USA).

## Results

### Genotyping of the schizophrenia sample

The allelic frequency of the disease-associated allele in our sample was 27.7%. 76 patients [52.1%] were homozygous for the wildtype (T/T), 59 [40.4 %] were heterozygous (T/C) and 11 [7.5%] were homozygous for the C-allele (C/C). The observed genotypic frequencies closely match the expected frequencies according to the Hardy-Weinberg principle (52.2%, 40.1% and 7.7% respectively) for the given allelic frequency.

### Clinical findings

We assessed the patients’ symptomatology, using the Bern Psychopathology Scale, which classifies symptoms into three different domains related to well-defined brain circuits (language, affective, and motor symptoms). We observed a significant effect of the number of C-alleles alleles on language symptoms (Wilks Lambda = 0.923; F(3,139) = 3.9; p-FDR = 0.033). The other symptom domains were not affected (all FDR-corrected *p*-values > 0.05). Post-hoc partial correlation analysis (corrected for gender and haloperidol equivalent dose) revealed that the SNP significantly affected quantitative language symptoms (*r* = −0.193; p-FDR = 0.015; df = 141) and qualitative language symptoms (*r* = −0.239; p-FDR = 0.006), but not subjective language symptoms (*r* = −0.027; p-FDR = 0.373). For both subcategories, patients with the genetic variant were more likely to be at the negative pole of the symptom axis, i.e., they exhibited speech-related negative symptoms, e.g., reduced spontaneity or pace of speech, reduced coherence of speech or frequent interruptions (see Supplementary Fig. 1).

### Neuroimaging

#### The effect of the GRIN2A variant resembles the differential effect of ketamine versus midazolam

**Fig. 2:**
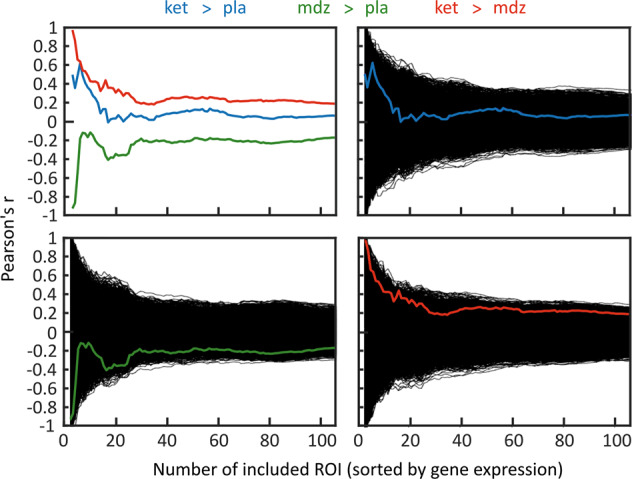
Correlation between functional connectivity changes associated with the genetic variant and the different pharmacological contrasts depending on the selected number of regions ranked by the expression of target genes. Connectivity changes related to the genetic variant (C-allele) are positively correlated with the contrasts ketamine > placebo as well as ketamine > midazolam and negatively correlated with the contrast midazolam > placebo. Absolute magnitude of correlation increases when iteratively removing regions with lower expression of the target genes. For the calculation of the p-values, we first created a null distribution of correlation curves and the respective areas under the curves (AUCs) by applying 10,000 random permutations of the genotype variable. Then we determined the percentage of AUCs exceeding (for the contrasts ketamine > placebo and ketamine > midazolam) or falling below (for the contrast midazolam > placebo) the observed AUC. The reported p-values reflect the respective percentages after FDR-correction. Figure 2b, c shows the performance of the correlation curve for each pharmacological contrast as compared to the curves generated by permutation of genotype. Considering the global AUC measure, only the contrasts ketamine > midazolam and midazolam > placebo reached statistical significance: AUC(ketamine > placebo) = 10.65; *p* = 0.188; AUC(midazolam > placebo) = −23.50; *p* = 0.023; AUC(ketamine > midazolam) = 28.58; *p* = 0.019 [all *p*-values FDR-corrected]. ket = ketamine; mdz = midazolam; pla = placebo; ROI = region of interest.

To assess the similarity between the effect of the GRIN2A variant on the one hand and the pharmacological effect of the NMDAR antagonist ketamine as well as the GABA-A receptor agonist Midazolam on the other hand, we conducted a correlation analysis using the averaged second level connectivity changes associated with the genetic variant and the respective pharmacological contrasts. Correlation curves revealed a positive correlation between the SNP effect and the effect of ketamine—both as compared to placebo and compared to midazolam (Fig. 2). In contrast, connectivity changes induced by midazolam were anti-correlated to the SNP-associated connectivity changes. The magnitude of correlation and anti-correlation, respectively, steadily increased when iteratively removing regions with lower common density of NMDARs, GABAergic, and glutamatergic neurons as measured by the post-mortem gene expression analysis. Highest absolute correlation coefficients were detected for the differential effect of ketamine as compared to midazolam as quantified by the absolute value of the area under the curve (AUC) which was significantly larger as compared to a null distribution of correlation curves obtained from 10,000 permutations of genotype within a Monte-Carlo simulation (AUC = 28.58; p-FDR = 0.019). The anti-correlation observed for the effect of midazolam vs. placebo was statistically significant, too (AUC = −23.50; p-FDR = 0.023). The positive correlation observed for the effect of ketamine > placebo did not reach statistical significance (AUC = 10.65; p-FDR = 0.188; see also Fig. 2 for further details).

#### Increased connectivity between the cuneus and caudate

**Fig. 3:**
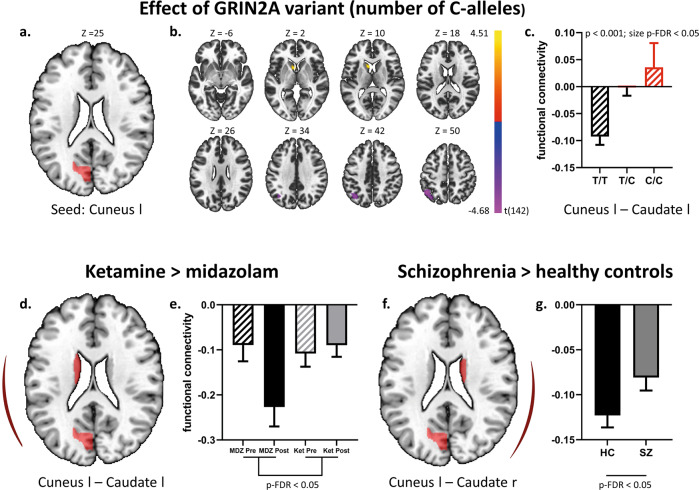
Hyper-connectivity between the cuneus and caudate. The impact of the genetic variant (C-allele) on the functional connectivity profile of the left cuneus was assessed using a seed-to-voxel-analysis (**a**–**c**). The location of the seed is demonstrated in **a**. Statistically significant clusters of hyper (left caudate)- or hypo-connectivity (left superior lateral occipital cortex) at a voxel-wise threshold of *p* < 0.001 and a cluster-size threshold of p-FDR < 0.05 are presented in **b**. Mean connectivity estimates (Fisher-z-transformed correlation coefficients) of the connectivity between the left cuneus and left caudate are depicted for each genotype using bar plots in **c**. The two connections of the cuneus which were affected by the genetic variant were subsequently assessed in the pharmaco-fMRI dataset (ketamine > midazolam, **d**, **e**) as well as the combined schizophrenia/control dataset (schizophrenia > healthy controls, **f**, **g**) applying ROI-to-ROI functional connectivity analyses. For the ketamine > midazolam contrast, we observed significant hyper-connectivity between the left cuneus and the left caudate, too, which was mainly explained by more negative connectivity estimates emerging after midazolam application. For the schizophrenia > healthy controls contrast we did not observe any significant group differences for the two connections and therefore also assessed the connections of the left cuneus with the corresponding contralateral (i.e. right) regions which indeed revealed a significant hyper-connectivity with the right caudate. Error bars represent the standard error of the mean. l left; r right.

Since the left cuneus was identified as the region with the highest averaged expression of the three target genes (see Supplementary Table 3), we studied the impact of the genetic variant on its functional connectivity profile using a seed to voxel analysis (Fig. 3a–c). The disease-associated variant was associated with increased functional connectivity of this seed with the left caudate and decreased functional connectivity with a cluster comprising the left superior lateral occipital cortex (sLOC) and the left angular gyrus. A significant hyper-connectivity between the left cuneus and the left caudate was also observed for the contrast ketamine > midazolam in the pharmaco-fMRI dataset, as revealed by a ROI-to-ROI functional connectivity analysis (Fig. 3d, e). This finding was explained by a tendency towards less negative connectivity estimates after ketamine application and more negative estimates after midazolam application with the latter observation dominating the overall effect (Fig. 3e). Within the schizophrenia group, the significant hyper-connectivity between the left cuneus and the left caudate associated with the genetic variant was explained by less negative connectivity estimates in an allele-dose dependent manner with homozygous allele carriers exhibiting even a positive mean connectivity estimate. Next, we wanted to determine whether these connectivity changes would be also associated with schizophrenia in general, comparing all patients with healthy control subjects. Since we did not detect any significant group differences for the two identified connections, we also tested the connections of the left cuneus with the contralateral (i.e. right) caudate and right superior lateral occipital cortex, applying a ROI-to-ROI analysis. Indeed, patients with schizophrenia exhibited a hyper-connectivity (reduced negative connectivity) between the left cuneus and the right caudate (for detailed statistics see Supplementary Table 6). Thus, hyper-connectivity between the cuneus and the caudate emerged as a common finding related to genetically and pharmacologically induced NMDA receptor dysfunction as well as schizophrenia.

#### Increased striato-pallido-thalamo-cortical connectivity

**Fig. 4:**
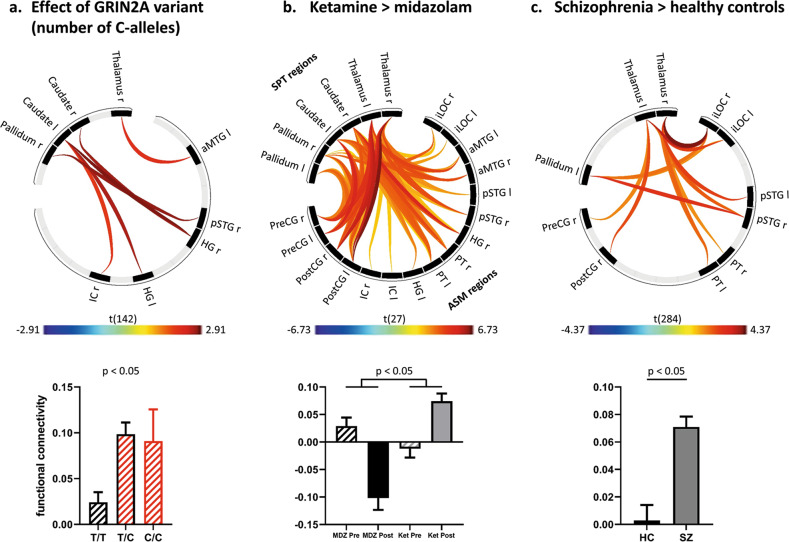
Striato-pallido-thalamo-cortical hyper-connectivity. Functional network connectivity analysis assessing the impact of the genetic variant (**a**), the differential effect of ketamine versus midazolam (**b**) or schizophrenia versus controls (**c**) on the overall connectivity between a striato-pallido-thalamic (SPT) and an auditory-sensory-motor (ASM) set of regions. A significant overall hyper-connectivity between the two sets of regions emerged for all three contrasts of interest. ROI-to-ROI connectivities that contributed most to the increase of overall network connectivity (as defined by an uncorrected *p*-value of < 0.05) are depicted in connectivity rings (upper panels). Their respective means and standard errors for each group / condition are illustrated in bar plots (lower panels). iLOC inferior lateral occipital cortex, aMTG anterior middle temporal gyrus, pSTG posterior superior temporal gyrus; HG Heschl’s gyrus, PT planum temporale, IC insular cortex, PostCG postcentral gyrus, PreCG precentral gyrus, l left, r right, Ket ketamine, MDZ midazolam, HC healthy controls, SZ schizophrenia.

Significant hyper-connectivity emerged between the striato-pallido-thalamic regions and ASM network for the genetic, pharmacological, and disease-related contrast, respectively. Among the different contrasts of interest, the pharmacological contrast exhibited the most pronounced and widespread connectivity changes (Fig. 4) with ketamine increasing and midazolam decreasing connectivity, respectively. For the genetic effect, we observed a substantial increase of connectivity for the heterozygous allele-carriers which did not further increase for the homozygous allele carriers (for further statistical details see Supplementary Table 7).

#### Decreased connectivity between an auditory (superior temporal gyrus) and visual (temporo-occipital) network

**Fig. 5:**
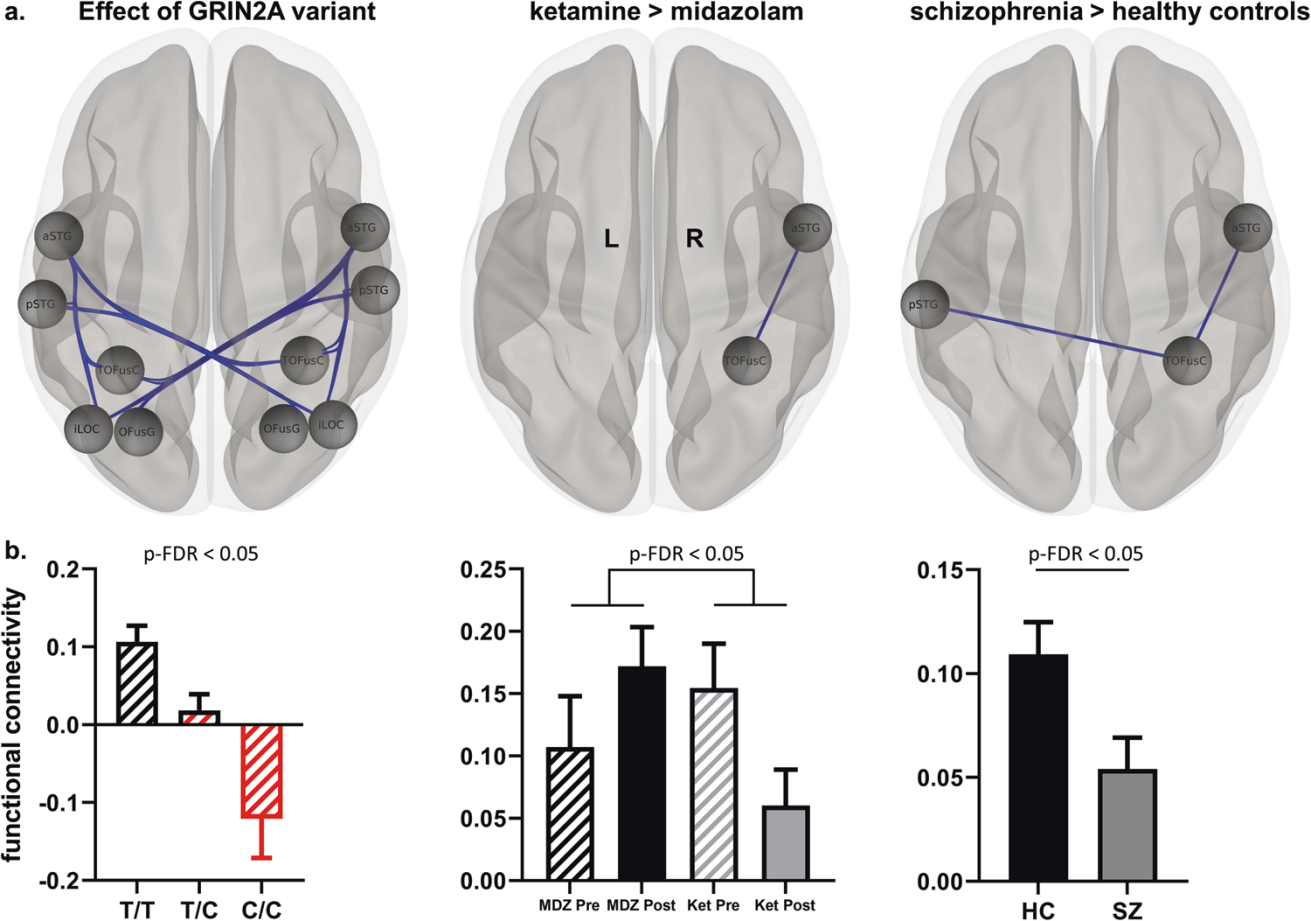
Reduced connectivity between auditory and visual regions. A hypothesis-free whole-brain functional network connectivity analysis revealed that the genetic variant was associated with reduced functional connectivity between an auditory (superior temporal) and visual (temporo-occipital) network (**a**, left panel). For visualization, all impaired ROI-to-ROI connections with an uncorrected *p*-value < 0.05 are depicted as blue lines connecting the respective ROIs. Among those, the two connections exhibiting the strongest effect (aSTGr-TOFusCr and pSTGl – TOFusCr) were subsequently tested in the pharmaco-fMRI dataset using the contrast ketamine > midazolam (**a**, middle panel) and in the combined schizophrenia/control dataset using the contrast schizophrenia > healthy controls (**a**, right panel). For the aSTGr-TOFusCr connection which was significantly affected in all three contrasts, the respective means and standard errors for each group/condition are illustrated as bar plots in the lower panels (**b**). aSTG anterior superior temporal gyrus, pSTG posterior superior temporal gyrus, TOFusC temporo-occipital fusiform cortex, OFusG occipital fusiform gyrus, iLOC inferior lateral occipital cortex, L left; R right, Ket ketamine, MDZ midazolam, HC healthy controls, SZ schizophrenia.

Subsequently, connectivity alterations associated with the genetic variant were assessed in a hypothesis-free manner, investigating functional network connectivity based on a whole-brain ROI-to-ROI analysis including all 106 regions of the Harvard Oxford Atlas. We identified reduced connectivity between a network comprising the bilateral anterior and posterior superior temporal gyrus (aSTG, pSTG) and a temporo-occipital network comprising the bilateral inferior lateral occipital cortex (iLOC), the bilateral occipital fusiform gyrus (OFusG) as well as the bilateral temporal occipital fusiform cortex (TOFusC)): F(4,139) = 6.54; p-FDR = 0.016. (See Fig. 5 and Supplementary Table 8).

Between these two networks, the two strongest reductions of connectivity were observed for the connection between the right anterior superior temporal gyrus and the right temporal occipital fusiform cortex (aSTG r – TOFusC r) as well as between the left posterior superior temporal gyrus and –again- the right occipital fusiform cortex (pSTG l – TOFusC r). Connectivity estimates gradually decreased with growing numbers of C-alleles and turned negative for the homozygous C/C-genotype (Fig. 5b).

We subsequently tested these two connectivities for the comparison of ketamine versus midazolam in the pharmaco-fMRI dataset and for the comparison of the whole schizophrenia sample versus the healthy control subjects. For the former comparison (ketamine > midazolam), we observed significantly reduced connectivity between the right anterior superior temporal gyrus and the right temporal occipital fusiform cortex (aSTG r – TOFusC r), too, which was explained by the opposing effects of the two drugs, i.e., a ketamine-induced decrease and a midazolam induced increase of functional connectivity. For the latter comparison (schizophrenia > healthy controls) we could reproduce reduced connectivity for both connections (aSTG r – TOFusC r and pSTG l – TOFusC r) in the patients. Accordingly, hypo-connectivity between the superior temporal gyrus and inferior temporo-occipital regions emerged as a consistent finding across the different datasets and contrasts relating it both to schizophrenia and NMDAR dysfunction.

### Correlation between functional connectivity and language symptoms

**Fig. 6:**
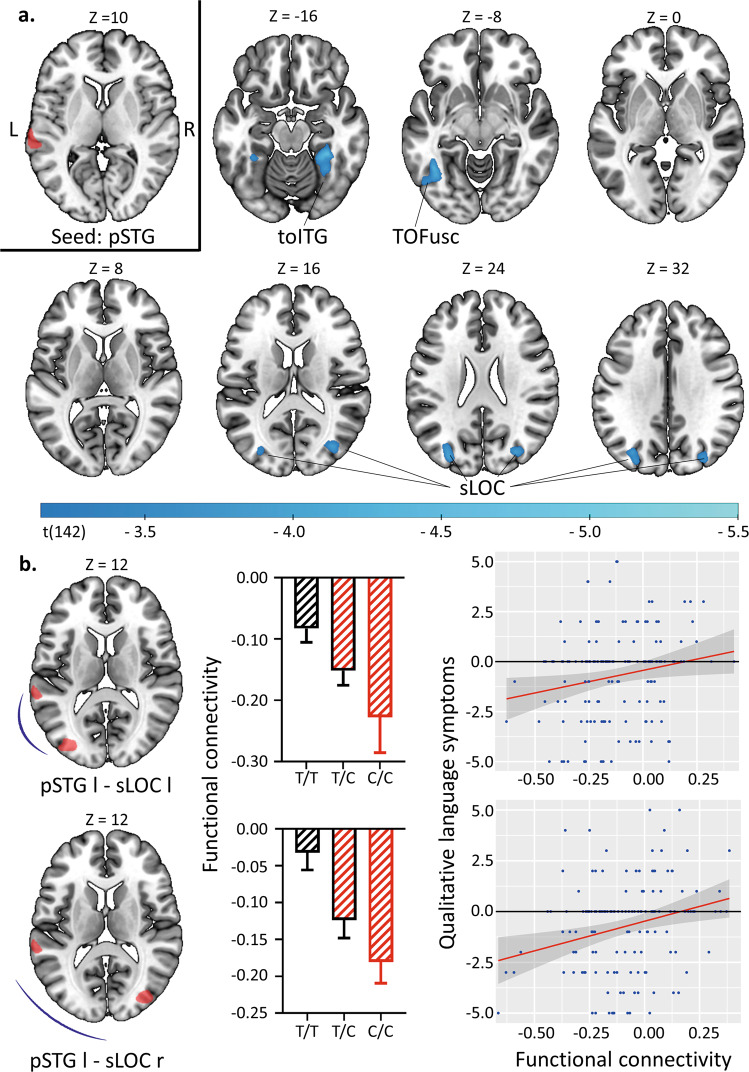
Exploring neural correlates of language symptoms associated with the GRIN2A variant. Statistical parametric maps obtained from a seed-to-voxel-analysis of the left posterior superior temporal gyrus (pSTG, Wernicke’s area) showing clusters of decreased connectivity associated with the genetic variant (C-allele) at a voxel-wise threshold of *p* < 0.001 and a cluster-size threshold of p-FDR < 0.05 (**a**). Among the four different connections affected by the genetic variant, two were significantly correlated with qualitative language symptoms of the Bern psychopathology scale (**b**). Error bars represent the standard error of the mean. pSTG posterior superior temporal gyrus, toITG temporo-occipital inferior temporal gyrus, TOFusC temporo-occipital fusiform cortex, sLOC superior lateral occipital cortex, l left, r right.

Whereas we could not detect significant connectivity changes related to the genetic variant for the inferior frontal gyrus seed, for the left posterior superior temporal gyrus, four clusters emerged comprising the bilateral superior occipital cortex (sLOC), the right temporo-occipital fusiform cortex (TOFusC r) and the left temporo-occipital inferior temporal gyrus (toITG l), respectively (Fig. 6). We subsequently assessed the association of this connectivity with qualitative language symptoms, as this language domain was most affected by the genetic variant. Indeed, it turned out that the language symptomatology correlated with the connectivity between the left superior temporal gyrus and the bilateral superior occipital cortex, i.e., the lower the connectivity estimate, the higher the burden of language-related negative symptomatology (left superior occipital cortex: *r* = 0.183, p-FDR = 0.028, *N* = 145; right superior occipital cortex: *r* = 0.254; p-FDR = 0.004, *N* = 145). The two other connections which were affected by the genetic variant did not show a significant correlation with the symptomatology (pSTG l – TOFusC r: *r* = 0.038; *p* = 0.325; pSTG l – toITG l: *r* = 0.097; *p* = 0.168). We subsequently tested the connections of the left posterior superior temporal gyrus with the bilateral superior lateral occipital cortex for the contrast ketamine > midazolam in the pharmaco-fMRI dataset which revealed a trend-level effect for the left superior occipital cortex (t(27) = −1.69; p-FDR = 0.104) only (see Supplementary Table 9). The comparison of the whole schizophrenia sample with the healthy control sample revealed a trend-level effect for both connections (pFDR = 0.069 for both connections, see Supplementary Table 9).

## Discussion

The present study assessed shared functional connectivity profiles of pharmacologically and genetically based NMDAR dysfunction by integrating resting state-fMRI data from 146 genotyped patients with schizophrenia and 142 healthy controls, pharmaco-fMRI data from 28 healthy participants, and postmortem whole-brain microarray gene expression data from 6 healthy donors.

The schizophrenia-related genetic variant of the NMDAR 2A subunit was associated with functional connectivity changes most resembling the differential pharmacological effect of ketamine versus midazolam. Importantly, this contrast has been shown to better imitate the functional connectivity changes observed in schizophrenia as compared to the more frequently studied difference between ketamine and placebo [16]. Moreover, the difference between ketamine and midazolam is of particular pathophysiological interest as it removes the common unspecific effects of both anesthetics, while emphasizing the diverging effects related to excitation (ketamine) and inhibition (midazolam) of glutamatergic pyramidal cells. Consequently, the observed connectivity changes may indicate that the common GRIN2A risk variant indeed promotes the excitatory/inhibitory imbalance in schizophrenia.

The resemblance between connectivity changes related to the genetic variant and the differential effect of ketamine versus midazolam became more pronounced when considering regions with high density of NMDARs, glutamatergic neurons, and parvalbumin-positive interneurons as suggested by the post-mortem gene expression dataset. In our mechanistic model, these regions should indeed reflect E/I imbalance related to hypofunction of NMDARs on parvalbumin-positive interneurons most directly. In this context, we chose GRIN1 instead of GRIN2A as one of our target genes, as GRIN2A expression may be less reliably estimated due to stronger age dependence [30, 31]. In our additional exploratory analysis (see Supplementary Results X), we indeed observed a weaker resemblance between the genetic and the ketamine > midazolam effect when choosing GRIN2A instead of GRIN1, although it was formally still significant.

From a more functional perspective, shared connectivity changes included hyper-connectivity between the basal ganglia/thalamus and an auditory-sensory-motor network as well as hypo-connectivity between auditory and visual regions. Increased functional connectivity between the thalamus and the auditory-sensory-motor (ASM) network is one of the most consistent findings which are shared by resting state-fMRI studies on schizophrenia and pharmaco-fMRI studies using ketamine [18–21]. Moreover, this imaging phenotype is also detected at different stages of the disease and in persons at high risk of schizophrenia [33–35]. Importantly, in clinical high-risk subjects, this pattern was even more pronounced in subjects who later developed schizophrenia [35]. The present study revealed that the extension of ASM network-thalamic hyper-connectivity to the basal ganglia which was recently demonstrated in patients with schizophrenia [21] is also characteristic for ketamine. Matching our hypothesis of an underlying E/I-imbalance, midazolam showed the opposite effect. Moreover, the schizophrenia-related GRIN2A variant exhibited a similar pattern suggesting that this typical imaging phenotype of schizophrenia may indeed be aggravated by a genetically driven dysfunction of NMDAR receptors. Hyper-connectivity within the cortico-striato-pallido-thalamo-cortical loops in patients with schizophrenia and ketamine models has been most consistently identified for regions belonging to the ASM network, i.e. particularly the bilateral auditory cortex, pre- and postcentral gyrus [19–21, 35]. However, some studies suggest that other sensory regions, especially visual regions may show a similar pattern [18, 33]. Accordingly, the here reported hyper-connectivity between the cuneus and the caudate which was identified for all contrasts of interest may add to this growing body of evidence.

Hypo-connectivity between auditory and visual regions mainly involved the superior temporal gyrus on the one hand and the fusiform and lateral occipital cortex on the other hand. Basic auditory processing deficits as assessed by behavioral, EEG or fMRI experiments, represent a consistent finding in patients with schizophrenia which may contribute to auditory hallucinations, higher-order cognitive deficits, and particularly language dysfunction [36–39]. On a macroscale level, they have been linked to structural and functional disturbances of the superior temporal gyrus, although recent findings suggest that some auditory deficits such as disturbed auditory change detection may even arise at early subcortical stages of the auditory pathway such as the inferior colliculus and thalamus [40]. On a microscale level, these deficits have indeed been assigned to dysfunction of NMDARs given the fact that they can be induced in healthy subjects by NMDAR antagonists, whereas other neurotransmitter receptors seem to be less important [38, 41, 42].

Besides pure activation deficits to different auditory stimulation paradigms in patients [39, 40, 43], ultra-high risk subjects [44, 45], and unaffected relatives [46] there is also a large body of evidence for disturbed functional connectivity of the superior temporal gyrus in schizophrenia [39, 40, 47–49]. Among different potential mechanisms, dysconnectivity in schizophrenia may also arise from NMDAR dysfunction, as it may be related to deficits of NMDAR-dependent synaptic plasticity [50, 51]. Altered functional connectivity profiles in schizophrenia may lead to an aberrant modular structure of the connectome which was most pronounced for sensory processing regions [52]. Moreover, abnormal modular connectome organization in subjects at clinical high risk for psychosis was shown to be associated with conversion to psychosis and the superior temporal gyrus emerged as one of the regions which were most abnormal in terms of their modular assignment [53]. Reduced connectivity between temporal/auditory and occipital/visual areas as demonstrated for the genetic variant, the pharmacological effect of ketamine versus midazolam as well as the comparison between schizophrenia and controls, was also detected in another study both in patients with first episode and chronic schizophrenia [54]. Notably, the respective reductions of connectivity were associated with a higher temporal variability of the functional connectivity of these regions which may indicate “decreased stability of attractor networks related to sensory processing”. Among the different temporo-occipital connections affected by the genetic variant in our study, the connection between the superior temporal gyrus and the fusiform cortex exhibited the strongest effect. Interestingly, this connection also emerged as one of the top features for the discrimination between patients with schizophrenia and healthy controls in a machine learning study [55].

On a behavioral level, the schizophrenia-related GRIN2A variant was associated with language-related negative symptomatology. Importantly, language dysfunction is considered a disease defining symptom both of schizophrenia and disorders related to exonic variants of GRIN2A (epilepsy-aphasia spectrum disorders) [13]. In the latter, language symptomatology is heterogeneous ranging from subtly impaired intelligibility of conversational speech to severe dysarthria, speech dyspraxia, receptive or expressive language delay/regression [13].Among the different functional connections of the left posterior superior temporal gyrus which were affected by the genetic variant, two connections correlated with the observed speech phenotype comprising the bilateral superior lateral occipital cortex. This region has been assigned to a syntactic processing network [56] and is connected to the former region (on the ipsilateral side) by the middle longitudinal fascicle [57]. Indeed, a previous study reported that the integrity of this fiber tract was affected in patients with schizophrenia which was related to disorganized speech in the patients [58]. Future studies may address whether the functional connectivity deficit observed in the C-allele carriers may be mediated by structural deficits of this fiber tract by applying diffusion tensor imaging. In the pharmaco-fMRI dataset, the differential of contrast of ketamine versus midazolam only had a trend-level effect and no behavioral data related to language symptomatology were available. Nevertheless, similar behavioral findings were observed in other pharmacological studies on NMDAR antagonists [59, 60]. Importantly, language deficits in schizophrenia may contribute to cognitive deficits, problems in social interaction and accordingly have a negative impact on employment status [61]. Therefore, the development of treatments successfully targeting language symptoms in schizophrenia is required. The findings of the present study may suggest that pharmacological treatment strategies enhancing NMDAR functioning (such as the promising glycine reuptake inhibitor BI 425809 [62]) might be beneficial for this symptom domain. In this context, it would be particularly interesting to determine whether the benefits are more pronounced for patients who are allele carriers of the GRIN2A-variant or exhibit a strong manifestation of the associated connectivity-changes.

## Conclusion

Given their genetic basis, their association with schizophrenia and their mechanistic implication suggested by the comparison with the pharmaco-fMRI dataset, we consider the here reported alterations of functional connectivity as potential endophenotypes of NMDAR dysfunction and E/I-imbalance in schizophrenia. These endophenotypes may help to identify patients with a common pathology of this molecular pathway, thus, providing a potential target for a personalized therapy.

## Supplementary information

Supplementary Material

Supplementary Figure 1

Supplementary Figure 2

Supplementary Figure 3

Supplementary Figure 4

## Data Availability

Computer code which was used for the analysis can be obtained from the authors upon reasonable request.
