# Supplementary material for: Functional connectivity signatures of NMDAR dysfunction in schizophrenia—integrating findings from imaging genetics and pharmaco-fMRI
Source: Transl Psychiatry. 2023 Feb 16;13:59. doi: 10.1038/s41398-023-02344-2 (PMC9935542; doi:10.1038/s41398-023-02344-2)
Supplement: Supplementary file 1 — Supplementary Material [file 41398_2023_2344_MOESM1_ESM.doc]

**Supplementary information**

**Content of this document**:

| Supplementary methods M1 | - Participants |
| --- | --- |
| Supplementary methods M2 | - Genotyping |
| Supplementary methods M3 | - Clinical assessment |
| Supplementary methods M4 | - Postmortem gene expression dataset |
| Supplementary methods M5 | - MRI data acquisition |
| Supplementary methods M6 | - fMRI-preprocessing |
| Supplementary methods M7 | -Assessment of striato-pallido-thalamo-cortical hyper-  connectivity |
| Supplementary methods M8 | - Hypothesis-free functional network connectivity analysis |
| Supplementary Results R1 | - Quantitative trait loci (QTL) analysis |
| Supplementary results R2 | - Replacement of GRIN1 by GRIN2A as a marker gene |
| Supplementary Results R3 | - Treating genotype as a categorical variable |
| Suppl. figure legends |  |
| Suppl. Tables 1-13 |  |
| References |  |
|  |  |
| **Separate documents:** | Supplementary Figures 1-4 |

**Supplementary Methods M1– Participants**

Schizophrenia sample and healthy control sample: Neuroimaging, genetic and clinical data were obtained from 146 in- and out-patients suffering from schizophrenia. Patients were recruited within the framework of an interventional brain imaging study conducted at the Department of Psychiatry, Psychotherapy and Psychosomatics of RWTH Aachen University Hospital and four academically associated regional psychiatric hospitals (Alexianer Krankenhaus, Aachen; ViaNobis Gangelt; LVR Klinik Langenfeld, LVR Klinikum Düsseldorf). The study was originally designed as a randomized controlled trial comparing the effect of different antipsychotic treatment strategies on brain volume (NCT02435095). All data presented here were obtained before any randomization or intervention. The study employed the following inclusion criteria: diagnosis of schizophrenia according to DSM-5, age of 18-65 years, written declaration of consent, subjects being contractually and mentally capable to attend the medical staffs' orders and MRI capability. Exclusion criteria comprised: relevant somatic diseases, which could have an impact on the conduct of the study based on clinical judgement of the treating physician (e.g. epilepsy, cancer), prior insufficiently documented drug therapy with antipsychotics, magnetic metals in or on the body, cardiac pacemakers and body piercings, pregnancy or lactation, hospitalization of the patient ordered by the court or public authorities, relationship of dependence or employment to sponsor or investigator and simultaneous participation in another clinical trial. The study protocol was approved by the ethics committee of the North Rhine medical association (AEKNO) and by the local regulatory authority of RWTH Aachen University Hospital (EK 156/16). Written informed consent was obtained from all participants, following a complete description of the study. Diagnosis of schizophrenia was confirmed according to DSM 5 criteria by trained clinical psychiatrists using the structured clinical interview for DSM disorders (SCID). Furthermore, symptomatology was assessed using the Bern Psychopathology Scale^1, 2^ for all but one patient who quit the study prematurely. Sociodemographic and clinical characteristics of the sample are provided in Supplementary Table 1. The intended sample size with respect to the primary outcome (gray matter change) of 574 patients could not be achieved and therefore the trial was stopped. To link the obtained resting state-fMRI connectivity findings of the genetic variant to the pathophysiology of schizophrenia, resting state fMRI data from 142 healthy control subjects were compared with the whole sample of patients suffering from schizophrenia. The healthy control subjects participated in different study arms of the same research network (APIC) approved by the local regulatory authority of RWTH Aachen University Hospital (EK 226/15, EK 050/17, EK 188/17, EK 059 /20) but no genetic information was available. Age and gender statistics of the healthy control sample and their comparison with the schizophrenia sample are provided in supplementary Table 2. Due to the common study background, the healthy control subjects had their MRI scans in the same MRI scanner as the patients and the same MRI sequences were used for the resting state and anatomical scans.” (for more details of the MRI sequence parameters see Supplementary Methods M5).

Pharmaco-fMRI sample: Thirty healthy male volunteers (average age: 27.3 ± 6.2 years, range: 19–37 years) recruited at the University of Auckland, participated in a single-blind, placebo-controlled, randomized pharmaco-fMRI study with a three-way cross-over design applying ketamine, midazolam and placebo. Each subject participated in all three sessions. Individual sessions were separated by 48 hours to allow for a wash-out of drug effects. Each session collected resting-state imaging data. Participants were instructed to have their eyes open and fixated on a small cross on a projection screen during infusion. Written informed consent was obtained from all participants and the local Ethics Committee approved the study (Central Health and Disability Ethics Committee Ref: 15/CEN/254). The data analyzed here have been also reported in previous publications^3,4,5^. Two participants were excluded from the analysis due to insufficient data quality. Accordingly, data from 28 subjects with three functional data sets each were eligible for the analysis. For the pharmaco-fMRI dataset, data for each of the three different sessions were split into the first and last 200 volumes (~ 7 min), i.e. pre-drug and post-drug infusion conditions, thus removing the 2-min bolus period.

**Supplementary Methods M2 - Genotyping**

EDTA blood samples obtained from each participant of the schizophrenia group were

genotyped for the SNP rs7191183 of the NMDA receptor 2A subunit (GRIN2A) by Sanger

Sequencing. Among the different SNPs of the GRIN2A gene which are associated with schizophrenia and in a tight linkage disequilibrium ^6-9^, we selected this SNP as it was considered the index SNP in the largest GWAS study^7^ at the time when we planned the analysis (2019). PCR conditions and primer sequences can be asked from the authors.

**Supplementary Methods M3 - Clinical assessment**

We assessed the patients’ symptomatology, applying the Bern Psychopathology Scale, which was developed to identify more homogeneous patient groups in the context of a system-oriented physiological model of schizophrenia The merit of this scale is founded in its inherent neurobiological plausibility assigning symptoms to three different domains related to well-defined brain circuits (language, affective, and motor symptoms). Those symptom domains are further subcategorized into quantitative, qualitative, and subjective (for the language and motor domain) or objective, subjective, and indirect symptoms (for the affective domain) and each symptom is rated on a bipolar axis (including a negative / hypoactive [-1], a positive / hyperactive [+1], and an average manifestation [0]). Finally the clinician assigns a global score to each domain on a 7 point scale ranging from -3 to 3 indicating no (0), mild (-1 / +1), moderate (-2 / +2) or severe (-3 / +3) symptom burden (for the respective results see Table 1). This neurobiologically driven symptom categorization and its bipolar axis differentiating between over- and hypoactive manifestations render the scale ideal for investigations on the relationship between neuroimaging and behavioral data. For a global assessment of symptom load within each domain, we also applied a multivariate general linear model to each domain using the sum scores of the respective symptom subcategories as the dependent variables and the number of disease-associated C-alleles as the independent variable. We corrected for effects of gender and antipsychotic medication load, by including these variables as predictors of no interest in the model. Antipsychotic medication load was quantified as haloperidol equivalent dose. Such equivalent doses were obtained by dividing the daily dose of each antipsychotic by its so called defined daily dose (DDD) and multiplying this ratio with the DDD of haloperidol^10^.

**Supplementary Methods M4 - Postmortem gene expression dataset**

The microarray-based gene expression data were downloaded from the website of the Allen Human Brain Atlas^11^ (<https://human.brain-map.org/>). This dataset contains gene expression data obtained from postmortem brains of 6 healthy donors between 18 and 68 years of age. RNA samples were extracted from about 400 anatomically annotated brain samples per hemisphere and analyzed using more than 62,000 gene probes. For two donors, data from both hemispheres were available. For the remaining four donors, only data from the left hemisphere were available. For background filtering, we utilized the binary indicator included in the Allen Brain dataset stating for each probe in each brain sample whether it measures an expression signal that exceeds background levels. We subsequently removed all probes which did not exceed the background in at least 50% of all samples across all donors. For a direct comparison with our functional connectivity results, we mapped the tissue samples of the Allen Human Brain Atlas to the brain parcellation of the FSL-Harvard-Oxford atlas which is implemented in the Conn matlab toolbox, by matching their Montreal Neurological Institute (MNI) coordinates. This atlas parcellates the cerebrum into 106 cortical and subcortical regions. Z-scores of gene expression levels of the target genes (see below) were averaged across samples mapping to the same region of the atlas, followed by renewed z-score calculation. Finally, the Z-values of each region of interest (ROI) were also averaged across donors and z-score calculation was repeated. Among the different probes targeting a single gene, we selected only those which showed the highest correlation with RNA-Sequencing results of the respective gene which were available for a subset of the tissue samples from two donors. Re-annotation of probes was done to ensure proper probe to gene mapping according to recent genetic knowledge^12^. “In order to assess between-donors variability for each probe, we calculated Fisher-z-transformed Pearson correlation coefficients for the regional gene expression profile for each pair of donors, averaged them across the six donors, and back-transformed these averages. Accordingly, we obtained Pearson correlation coefficients above 0.66 (p < 0.001) for each probe.

**Supplementary Methods M5 - MRI data acquisition**

Schizophrenia sample and healthy control sample: Magnetic resonance imaging was conducted using a 3 Tesla Tim Trio Scanner (Siemens, Erlangen, Germany) with a 20-channel head coil. Anatomical reference imaging applied a T1-weighted 3d sequence (MPRAGE, echo time= 3.03 ms; repetition time = 2,000 ms; inversion time = 900 ms; flip angle=9°; field of view =256x256 mm²; voxel size = 1x1x1 mm^3^; 176 sagittal partitions). Resting state-fMRI data were acquired using a T2*-weighted echo-planar imaging (EPI) sequence (echo time = 28ms; repetition time = 2000 ms; flip angle = 77°; field of view = 192x192 mm^2^; voxel size = 3x3x3 mm^3^; interleaved acquisition of 34 transverse slices); 240 volumes (~ 8 minutes). Quality assurance procedures were applied within 24h.

Pharmaco-fMRI sample: All measurements were conducted using a 3 T MRI scanner (Siemens Skyra, Erlangen, Germany) with a 20-channel head coil. A high-resolution MPRAGE scan was acquired in one of the three sessions for each participant (TR 2100 ms, TE 3.42 ms, flip angle 9°, 192 slices, voxel size 1 × 1 × 1 mm). Resting state-fMRI data were acquired using a T2*-weighted echo-planar imaging (EPI) sequence (repetition time = 2,200 ms, echo time = 27 ms, flip angle = 79°, 30 interleaved 3-mm slices, field of view = 215 x 215 mm^2^, voxel size 3 × 3 × 3 mm^3^). 437 volumes were acquired (7 min predrug, 9 min postdrug). Drugs were administered at a subanesthetic level through an intravenous line. Application rates were controlled by an infusion pump (Alaris PK, UK), programmed by a supervising anesthesiologist. Ketamine or Midazolam were administered with a bolus dose of 0.25 mg/kg or 0.03 mg/kg, followed by an infusion with a rate of 0.25 mg/kg/hour infusion or 0.03 mg/kg/hour, respectively. Doses were similar to those used in previous literature^13, 14^. Participants were monitored for their respiration and blood pressure during data acquisition.

**Supplementary Methods M6 - fMRI-preprocessing:**

All fMRI datasets were subjected to the default pre-processing pipeline as implemented in the conn toolbox including functional realignment and unwarping, slice-timing correction, outlier identification, direct segmentation and normalization to standard MNI space as well as spatial smoothing (8 mm full-width-at-half-maximum Gaussian kernel). Using ordinary least squares (OLS) regression, noise components originating from the cerebral white matter and cerebrospinal areas, head motion parameters, identified outlier scans as well as constant and first-order linear session effects were removed from the BOLD time series. Subsequently, a temporal band-pass filter of 0.008 to 0.09 Hz was applied to the time series.

**Supplementary Methods M7 – Assessment of striato-pallido-thalamo-cortical hyper-connectivity**

One of the most consistent resting state-fMRI findings which is shared both by patients with schizophrenia and human ketamine models of schizophrenia is a hyper-connectivity within cortico-striato-pallido-thalamo-cortical (CSPTC) loops, i.e. between the thalamus, striatum (particularly the caudate^15^) or pallidum on the one hand and the auditory-sensory-motor (ASM) network on the other hand. To assess the effect of the genetic variant, the differential effect of ketamine versus midazolam and the general effect of schizophrenia, respectively, on the overall connectivity between the two sets of regions (striato-pallido-thalamic and ASM network), we conducted a multivariate ROI to ROI analysis, using the functional network connectivity analysis implemented in the Conn toolbox^16^. Thereto, for each dataset or effect of interest, all corresponding ROI-to-ROI connectivity estimates reflecting connections between the two sets of regions entered a multivariate parametric general linear model analysis resulting in an F-statistic and an associated p-value. For the visualization of the corresponding ROI-to-ROI connections contributing most to the overall pattern of connectivity changes, we applied an uncorrected threshold of p<0.05 at the connection level, which is the default setting in the Conn toolbox. For the definition of the striato-pallido-thalamic set of regions, we used the bilateral thalamus, caudate and pallidum as defined by the Harvard Oxford Atlas. The selection of regions of the ASM network was based on the definition of Yeo et al^17^. Accordingly, we included all regions of the Harvard Oxford Atlas matching this definition, namely the bilateral Heschl‘s Gyrus, temporal plane, insular cortex, precentral and postcentral gyrus. Additional ROIs were defined based on a meta-analysis of thalamo-cortical dysconnectivity in patients with schizophrenia^18^. MNI coordinates of peak voxels identified by this meta-analysis were assigned to the respective regions of the Harvard Oxford Atlas which were then included -together with their bilateral counterparts - as ROIs in our connectivity analysis. These additional ROIs comprised the bilateral anterior middle temporal gyrus, posterior superior temporal gyrus and inferior lateral occipital cortex (see Supplementary Table 5 for further details).

**Supplementary Methods M8 – Hypothesis-free functional network connectivity analysis**

To identify further NMDAR-related imaging phenotypes, we applied another functional network connectivity analysis (see above) studying the effect of the GRIN2A variant on a whole-brain level and in a hypothesis-free manner. Networks of ROIs were defined using a data-driven hierarchical clustering procedure (complete-linkage clustering^19^) based on anatomical proximity and functional similarity metrics of ROIs. Subsequently, for each network, the within network connectivity and for each pair of networks, the between network connectivity were assessed, respectively. Thereto, for each within- or between-network connectivity, all corresponding ROI-to-ROI connectivity estimates entered a multivariate parametric general linear model analysis resulting in an F-statistic and an associated p-value. Only network connections whose p-values remained significant after FDR-correction (p-FDR < 0.05) were reported. For the visualization of the corresponding ROI-to-ROI connections, we applied an uncorrected threshold of p<0.05 at the connection level. Again, the two strongest connectivity changes related to the genetic variant were subsequently re-assessed in the pharmaco-fMRI dataset for the contrast ketamine > midazolam as well as in the combined schizophrenia / control sample for the contrast schizophrenia > healthy controls using ROI-to-ROI analyses.

**Supplementary Results R1- Quantitative trait loci (QTL) analysis**

Previous research suggests that the respective schizophrenia-associated intronic variants of GRIN2A promote DNA methylation at GRIN2A-related CpG-sites^20^ which may consequently lead to reduced GRIN2A expression. More precisely, promotion of DNA methylation was demonstrated by Niu et al. for the SNP rs9926303^20^. Notably, this is one of the SNPs which are in a tight linkage disequilibrium with the here reported SNP rs7191183 and -accordingly - associated with schizophrenia^9^. We complemented this research, querying Quantitative trait loci (QTL) databases. Indeed, we obtained further evidence for GRIN2A hypermethylation using Brain xQTLServe^21^ (<http://mostafavilab.stat.ubc.ca/xQTLServe>): The variant was associated with significantly increased DNA methylation at CpG sites cg08001123 (beta = 0.565; se = 0.035; p-FDR < 0.001), cg16783478 (beta = 0.377; se = 0.040; p-FDR < 0.001) and cg03616148 (beta = 0.236, se = 0.042, p-FDR < 0.001) all of which are located within the GRIN2A gene. As a complementary finding, the Brain xQTLServe resource revealed that the variant was also associated with a tendency to reduced histone acetylation in 20 out of 22 positions located within a range of ± 1 MB around the SNP. When restricting the analysis to positions within the gene only, one position remained statistically significant after FDR-correction for multiple testing (beta = -0.117; se = 0.042; p-FDR = 0.039). In line with this, the BrainSeq Phase 2 resource (http://eqtl.brainseq.org/phase2/)^22^ revealed that the variant was associated with a significantly reduced expression of GRIN2A transcripts (beta = -0.059; se = 0.010; p-FDR < 0.001) . Furthermore, the variant was associated with altered splicing (novel junctions) of the GRIN2A transcripts, in both the dorsolateral prefrontal cortex (DLPCF) (beta = 0.772, se = 0.053; p-FDR < 0.001) and the hippocampus region (beta = 0.725; se = 0.053; p-FDR < 0.001), which might indicate aberrant transcripts and -as a consequence - nonsense mediated decay (NMD) thus contributing to reduced overall transcript levels.

**Supplementary Results R2 – Replacement of GRIN1 by GRIN2A as a marker gene**

In our original analysis, we assessed the similarity between the effect of the SNP and each of the three pharmacological effects (ketamine > placebo, midazolam > placebo, ketamine > midazolam) on functional connectivity, using a correlation analysis for which regions were ranked according to their averaged expression (i.e. averaged z-values) of the three marker genes GRIN1, Parvalbumin and SLC17A7 (encoding the ‘vesicular glutamate transporter 1’ - VGLUT1) as revealed by the analysis of the Allen Brain dataset. Since GRIN1 encodes the constitutive NMDAR 1 subunit, the impact of genetic variants of GRIN2A are functionally only relevant if GRIN1 is co-expressed. Moreover, expression of GRIN2A shows a stronger age-dependence^23, 24^ and may consequently be less reliably estimated. For these reasons, we decided to choose GRIN1 instead of GRIN2A as a marker gene for our original analysis Nevertheless, we also conducted an exploratory analysis using GRIN2A instead of GRIN1, i.e. we ranked the regions according to their averaged gene expression of the marker genes GRIN2A, Parvalbumin as well as SLC17A7 and re-ordered the 2^nd^ level matrices of connectivity changes accordingly. As presented in the main manuscript, we repeatedly assessed the correlation between connectivity changes related to the genetic variant or the respective pharmacological contrasts, starting from the full 106 x 106 matrix of connectivity changes and iteratively removing the region with the lowest expression of the target genes until only the 3 x 3 submatrix of the highest expressing regions remained. On a descriptive level, the curve reflecting the correlation between the SNP effect and the effect of midazolam versus placebo showed a similar behavior as compared to our original analysis, i.e. the effects were anti-correlated and the absolute magnitude of anti-correlation steadily increased when iteratively removing regions with a lower average expression of the three target genes. In contrast, connectivity changes related to the contrast ketamine > placebo were positively correlated with the genetic effect only when including more than 13 regions. Thereafter, negative correlation coefficients were obtained and the curve further decreased until a local minimum was reached at a number of five regions. Subsequently, the curve increased again and turned positive for the final set of three regions. Accordingly, for the contrast ketamine > midazolam we observed a similar dip of the curve with a local minimum at a number of 7 regions whereas the curve strongly increased thereafter reaching a correlation coefficient of r = 0.90 at the final set of three regions. The absolute values of the respective areas under the curves were still significantly larger as compared to the null distribution for the contrasts ketamine > midazolam (AUC = 22.85; p = 0.030 [FDR-corrected]) and midazolam > placebo (AUC = -22.33; p = 0.030 [FDR-corrected]), whereas the contrast ketamine > placebo did not show a significant effect (AUC = 4.61; p = 0.354 [FDR-corrected]). Thus, when replacing GRIN1 by GRIN2A in this analysis, even though the magnitude of the effects became smaller, the positive correlation of the genetic effect with the pharmacological effect of ketamine versus midazolam as well as the negative correlation with the effect of midazolam versus placebo remained statistically significant.

**Supplementary Results R3 – Treating genotype as a categorical variable**

In our original analysis, we estimated the effect of the genetic variant on functional connectivity by using the number of minor alleles (0, 1 or 2) as a regressor. We chose this approach as we assumed an additive genetic model as commonly employed in Expression Quantitative Trait Locus (eQTL) analyses^25, 26^ and suggested by the findings obtained from our own eQTL database query (see Supplementary Results R1). In an additional exploratory analysis, we treated the genotype as a categorical variable differentiating between the absence (i.e. wildtype) and the presence of the minor allele (pooling homozygous and heterozygous allele carriers), while keeping the rest of model unchanged (gender and haloperidol equivalent doses were included as regressors of no interest). The respective correlation curves estimating the similarity between the effect of the genetic variant and the respective pharmacological contrasts closely resembled those derived from our original analysis (see Supplementary Figure 3 and Figure 2). When using our original set of target genes (GRIN1, Parvalbumin and SLC17A7), significant correlation or anticorrelation, respectively, was observed for the contrast ketamine > midazolam (AUC = 29.02; p = 0.014 ) as well as midazolam > placebo (AUC = -23.24; p = 0.025), whereas the contrast ketamine > placebo was not significant (AUC = 11.35; p = 0.185, see Supplementary Figure 3).

Similar results were also obtained for the modified set of target genes (i.e., GRIN2A, Parvalbumin and SLC17A7; compare supplementary results R2): AUC (ketamine > midazolam) = 25.21, p = 0.028; AUC (midazolam > placebo) = -22.44, p = 0.028; AUC (ketamine > placebo) = 7.42, p = 0.271 (see Supplementary Figure 4).

The seed to voxel-functional connectivity analysis of the left cuneus revealed qualitatively similar findings as compared to our original analysis, too: Again, two clusters exhibiting significant connectivity differences between the two groups emerged: A cluster of hyper-connectivity (i.e. higher connectivity for the pooled sample of homozygous and heterozygous allele carriers) was observed in the left caudate and a cluster of hypo-connectivity was observed in the left superior lateral occipital cortex. The number of voxels assigned to each cluster and peak coordinates minimally differed from the original findings (for detailed statistics see SupplementaryTable_10 ).

The corresponding functional network connectivity analysis of striato-pallido-thalamo-cortical connectivity revealed a significantly increased connectivity between the two sets of region, too (see SupplementaryTable_11). At the specified threshold used to identify the most contributing connections (p < 0.05 uncorrected) the number of connections emerging slightly increased as compared to our original analysis, while one connection fell above the threshold (aMTGl- Thalamus r).

The hypothesis-free functional network connectivity analysis of the genetic effect also revealed very similar findings as in our original analysis. Significantly reduced connectivity emerged between a network comprising the bilateral superior temporal gyrus (anterior and posterior division) and a temporo-occipital network comprising the bilateral inferior lateral occipital cortex (iLOC), the bilateral occipital fusiform gyrus (OFusG) as well as the bilateral temporal occipital fusiform cortex (TOFusC): F(4,139) = 6.92; p-FDR = 0.009. Only slight differences were observed concerning the statistics of individual connections leading to a minimally different set of connections falling below the uncorrected p-value-level of 0.05 (see Supplementary Table_12 ).

For the language network seeds, in contrast to the original analysis, we observed a significant cluster of hypo-connectivity for the IFG seed, too. This cluster comprised the left temporal pole (TP l) and the left anterior superior temporal gyrus (aSTG l). Note that, for the pSTG-seed, the analogue of the 4^th^ cluster located in the temporo-occipital inferior temporal cortex (toITG) presented in Supplementary Table 9 has become much larger, extending to the left inferior lateral occipital cortex (iLOC). Moreover, a corresponding cluster of hypo-connectivity is also found on the contralateral side (detailed statistics for both seed-to-voxel analyses are provided in Supplementary Table 13).

**Supplementary Figure legends**

**Supplementary Figure 1.** **Language disturbances related to NMDAR dysfunction**

1. Percentages of patients within each genotype (upper panels: wildtype, middle panels: heterozygous, lower panels: homozygous) rated as hypoactive / inhibited (-1), normal (0) and hyperactive / disinhibited (+1) for each of the items which are categorized into quantitative (left panels), qualitative (middle panels), and subjective symptoms (right panels). Inspection of the distributions reveals that the SNP is associated with the hypoactive / inhibited phenotype for most items within the quantitative and qualitative subcategory which becomes particularly apparent for the homozygous genotype. T/T, T/C, and C/C refer to the wildtype, heterozygous, and homozygous genotype, respectively.
2. Average sum scores for each language subcategory (quantitative, qualitative, and subjective symptoms) sorted by genotype. Error bars represent the standard error of the mean. FDR-corrected p-values refer to the correlation between the number of C- alleles and the respective sum score.

**Supplementary Figure 2**. Correlation curves assessing the similarity between the effect of the genetic variant and each of the three pharmacological contrasts on functional connectivity. In contrast to our original analysis, regions were ranked by their average expression of the genes GRIN2A, Parvalbumin as well as SLC17A7, i.e. GRIN1 was replaced by GRIN2A (for further details see the text of supplementary results R2). The absolute values of the respective areas under the curves were significantly larger as compared to the null distribution of correlation curves (black curves) for the contrasts ketamine > midazolam and midazolam > placebo, whereas the contrast ketamine > placebo did not show a significant effect.

**Supplementary Figure 3**. Correlation curves assessing the similarity between the effect of the genetic variant and each of the three pharmacological contrasts on functional connectivity. In contrast to our original analysis presented in Figure 2, we treated the genotype as a categorical variable differentiating between the absence (i.e. wildtype) and the presence of the minor allele (homozygous or heterozygous allele carriers)

**Supplementary Figure 4**. Correlation curves assessing the similarity between the effect of the genetic variant and each of the three pharmacological contrasts on functional connectivity. As a modification of the previous analyses, GRIN1 was replaced by GRIN2A as a target gene (compare Supplementary Figure 2) and we treated the genotype as a categorical variable (compare Supplementary Figure 3)

**Table 1.** Sociodemographic and clinical characteristics of the patients with schizophrenia

| Characteristic | | Wildtype (T/T)  (N = 76) | | Heterozygous (T/C)  (N = 59) | | Homozygous (C/C)  (N = 11) | | Comparison | |
| --- | --- | --- | --- | --- | --- | --- | --- | --- | --- |
|  | | Mean | SD | Mean | SD | Mean | SD | F(df = 2, 143) | p |
| Age (years) | | 33.4 | 11.6 | 30.8 | 10.5 | 32.7 | 14.6 | 0.88 | 0.418 |
| Education (years)^a^ | | 11.8 | 2.7 | 11.3 | 2.3 | 12.4 | 3.6 | 0.89 | 0.414 |
| Parental education (years)^a^ | | 11.5 | 3.0 | 12.4 | 3.0 | 13.0 | 3.7 | 2.12 | 0.124 |
| Duration of illness (years) | | 4.4 | 6.0 | 5.5 | 7.4 | 6.3 | 9.4 | 0.63 | 0.536 |
| Bern Psychopathology Scale | |  |  |  |  |  |  | Pearson’s r | p |
| Global score language | | -0.1 | 1.2 | -0.2 | 1.4 | -1.1 | 1.2 | -0.15 | 0.033 |
| Global score affectivity | | -1.1 | 1.0 | -0.9 | 1.1 | -1.5 | 0.8 | -0.02 | 0.773 |
| Global score motor behavior | | -0.2 | 0.9 | -0.4 | 0.8 | -0.6 | 1.1 | -0.13 | 0.065 |
|  | | Median | IQR | Median | IQR | Median | IQR | χ2 (df = 2)^b^ | p |
| Haloperidol equivalent dose (mg/day) | | 6.4 | 14.0 | 6.4 | 14.0 | 6.4 | 5.2 | 0.02 | 0.99 |
|  | |  |  |  |  |  |  |  |  |
|  | | N | % | N | % | N | % | χ2 (df = 2) | p |
| Gender | |  |  |  |  |  |  | 0.39 | 0.85 |
| Female | | 22 | 28.9 | 16 | 27.1 | 4 | 36.4 |  |  |
| Male | | 54 | 71.1 | 43 | 72.9 | 7 | 63.6 |  |  |
|  | |  |  |  |  |  |  |  |  |
| Smoking status | |  |  |  |  |  |  | 7.60 | 0.107 |
| Current smoker | | 42 | 55.3 | 39 | 67.2 | 5 | 45.5 |  |  |
| Ex-smoker | | 9 | 11.8 | 2 | 3.4 | 3 | 27.3 |  |  |
| Non-smoker | | 25 | 32.9 | 17 | 29.3 | 3 | 27.3 |  |  |
|  | |  |  |  |  |  |  |  |  |
| Current FGA-treatment | |  |  |  |  |  |  | 2.43 | 0.297 |
| yes | | 4 | 5.3 | 5 | 8.5 | 2 | 18.2 |  |  |
| no | | 72 | 94.7 | 54 | 91.5 | 9 | 81.8 |  |  |
|  | ^a^ Including formal 3-year job apprenticeship with compulsory 1 day/week school attendance and university education.  ^b^ Kruskal Wallis test was employed due to unequal variances and non-normal distributions. Equivalent doses were calculated using the defined daily dose^10^  df =degrees of freedom; SD = standard deviation; IQR = Interquartil range; FGA = first generation antipsychotic | | | | | | | | |

**Supplementary Table 2.** Age and gender statistics of the whole sample of patients with schizophrenia and healthy control subjects

| Characteristic | Schizophrenia  (N = 146) | | Healthy controls  (N = 142) | | Comparison | |
| --- | --- | --- | --- | --- | --- | --- |
|  | Mean | SD | Mean | SD | t (df = 286) | p |
| Age (years) | 32.3 | 11.37 | 30.2 | 11.79 | 1.55 | 0.122 |
|  |  |  |  |  |  |  |
|  | N | % | N | % | χ2 (df = 1) | p |
| Gender |  |  |  |  |  |  |
| Female | 42 | 28.8 | 54 | 38.0 | 2.78 | 0.096 |
| Male | 104 | 71.2 | 88 | 62.0 |  |  |
|  |  |  |  |  |  |  |
| SD = standard deviation; df = degrees of freedom | | | | | | |

| **Supplementary Table 3.** Gene expression-based ranking of atlas regions | |
| --- | --- |
| **Harvard Oxford Atlas Region** | **Z-value** |
| atlas.Cuneal l (Cuneal Cortex Left) | 0.94 |
| atlas.IFG oper r (Inferior Frontal Gyrus, pars opercularis Right) | 0.84 |
| atlas.AG r (Angular Gyrus Right) | 0.75 |
| atlas.IFG oper l (Inferior Frontal Gyrus, pars opercularis Left) | 0.72 |
| atlas.SPL l (Superior Parietal Lobule Left) | 0.72 |
| atlas.SFG r (Superior Frontal Gyrus Right) | 0.71 |
| atlas.CO l (Central Opercular Cortex Left) | 0.71 |
| atlas.PostCG r (Postcentral Gyrus Right) | 0.71 |
| atlas.PreCG l (Precentral Gyrus Left) | 0.70 |
| atlas.iLOC l (Lateral Occipital Cortex, inferior division Left) | 0.70 |
| atlas.ICC l (Intracalcarine Cortex Left) | 0.70 |
| atlas.IFG tri l (Inferior Frontal Gyrus, pars triangularis Left) | 0.69 |
| atlas.OP l (Occipital Pole Left) | 0.68 |
| atlas.PO l (Parietal Operculum Cortex Left) | 0.68 |
| atlas.pSTG r (Superior Temporal Gyrus, posterior division Right) | 0.68 |
| atlas.AG l (Angular Gyrus Left) | 0.67 |
| atlas.PO r (Parietal Operculum Cortex Right) | 0.67 |
| atlas.PreCG r (Precentral Gyrus Right) | 0.67 |
| atlas.IFG tri r (Inferior Frontal Gyrus, pars triangularis Right) | 0.66 |
| atlas.PostCG l (Postcentral Gyrus Left) | 0.64 |
| atlas.pSMG r (Supramarginal Gyrus, posterior division Right) | 0.63 |
| atlas.aMTG r (Middle Temporal Gyrus, anterior division Right) | 0.61 |
| atlas.MidFG l (Middle Frontal Gyrus Left) | 0.60 |
| atlas.aSMG l (Supramarginal Gyrus, anterior division Left) | 0.59 |
| atlas.pSMG l (Supramarginal Gyrus, posterior division Left) | 0.59 |
| atlas.sLOC r (Lateral Occipital Cortex, superior division Right) | 0.58 |
| atlas.pITG r (Inferior Temporal Gyrus, posterior division Right) | 0.56 |
| atlas.pSTG l (Superior Temporal Gyrus, posterior division Left) | 0.55 |
| atlas.PaCiG r (Paracingulate Gyrus Right) | 0.54 |
| atlas.toITG l (Inferior Temporal Gyrus, temporooccipital part Left) | 0.54 |
| atlas.SFG l (Superior Frontal Gyrus Left) | 0.54 |
| atlas.SCC l (Supracalcarine Cortex Left) | 0.53 |
| atlas.sLOC l (Lateral Occipital Cortex, superior division Left) | 0.53 |
| atlas.OFusG l (Occipital Fusiform Gyrus Left) | 0.51 |
| atlas.HG l (Heschls Gyrus Left) | 0.50 |
| atlas.MidFG r (Middle Frontal Gyrus Right) | 0.50 |
| atlas.FP l (Frontal Pole Left) | 0.48 |
| atlas.FP r (Frontal Pole Right) | 0.48 |
| atlas.FOrb r (Frontal Orbital Cortex Right) | 0.47 |
| atlas.pMTG l (Middle Temporal Gyrus, posterior division Left) | 0.47 |
| atlas.SMA L(Supplementary Motor Cortex- Left) | 0.47 |
| atlas.aMTG l (Middle Temporal Gyrus, anterior division Left) | 0.47 |
| atlas.FOrb l (Frontal Orbital Cortex Left) | 0.46 |
| atlas.toITG r (Inferior Temporal Gyrus, temporooccipital part Right) | 0.45 |
| atlas.pMTG r (Middle Temporal Gyrus, posterior division Right) | 0.44 |
| atlas.SPL r (Superior Parietal Lobule Right) | 0.44 |
| atlas.iLOC r (Lateral Occipital Cortex, inferior division Right) | 0.43 |
| atlas.PaCiG l (Paracingulate Gyrus Left) | 0.41 |
| atlas.PT r (Planum Temporale Right) | 0.40 |
| atlas.Precuneous (Precuneous Cortex) | 0.40 |
| atlas.aSTG r (Superior Temporal Gyrus, anterior division Right) | 0.40 |
| atlas.FO l (Frontal Operculum Cortex Left) | 0.39 |
| atlas.toMTG l (Middle Temporal Gyrus, temporooccipital part Left) | 0.39 |
| atlas.TOFusC r (Temporal Occipital Fusiform Cortex Right) | 0.38 |
| atlas.pITG l (Inferior Temporal Gyrus, posterior division Left) | 0.38 |
| atlas.CO r (Central Opercular Cortex Right) | 0.38 |
| atlas.toMTG r (Middle Temporal Gyrus, temporooccipital part Right) | 0.38 |
| atlas.LG r (Lingual Gyrus Right) | 0.34 |
| atlas.HG r (Heschls Gyrus Right) | 0.34 |
| atlas.aITG l (Inferior Temporal Gyrus, anterior division Left) | 0.32 |
| atlas.FO r (Frontal Operculum Cortex Right) | 0.32 |
| atlas.PC (Cingulate Gyrus, posterior division) | 0.29 |
| atlas.OP r (Occipital Pole Right) | 0.27 |
| atlas.PP r (Planum Polare Right) | 0.27 |
| atlas.aSTG l (Superior Temporal Gyrus, anterior division Left) | 0.26 |
| atlas.Cuneal r (Cuneal Cortex Right) | 0.26 |
| atlas.MedFC (Frontal Medial Cortex) | 0.23 |
| atlas.OFusG r (Occipital Fusiform Gyrus Right) | 0.22 |
| atlas.SMA r (Supplementary Motor Cortex- Right) | 0.22 |
| atlas.ICC r (Intracalcarine Cortex Right) | 0.21 |
| atlas.pTFusC r (Temporal Fusiform Cortex, posterior division Right) | 0.19 |
| atlas.TP l (Temporal Pole Left) | 0.16 |
| atlas.LG l (Lingual Gyrus Left) | 0.15 |
| atlas.aTFusC l (Temporal Fusiform Cortex, anterior division Left) | 0.11 |
| atlas.pTFusC l (Temporal Fusiform Cortex, posterior division Left) | 0.10 |
| atlas.aITG r (Inferior Temporal Gyrus, anterior division Right) | 0.06 |
| atlas.SCC r (Supracalcarine Cortex Right) | 0.05 |
| atlas.PT l (Planum Temporale Left) | 0.02 |
| atlas.aSMG r (Supramarginal Gyrus, anterior division Right) | 0.00 |
| atlas.aTFusC r (Temporal Fusiform Cortex, anterior division Right) | -0.01 |
| atlas.IC r (Insular Cortex Right) | -0.04 |
| atlas.PP l (Planum Polare Left) | -0.06 |
| atlas.TOFusC l (Temporal Occipital Fusiform Cortex Left) | -0.06 |
| atlas.TP r (Temporal Pole Right) | -0.12 |
| atlas.IC l (Insular Cortex Left) | -0.14 |
| atlas.AC (Cingulate Gyrus, anterior division) | -0.20 |
| atlas.pPaHC l (Parahippocampal Gyrus, posterior division Left) | -0.25 |
| atlas.aPaHC l (Parahippocampal Gyrus, anterior division Left) | -0.34 |
| atlas.pPaHC r (Parahippocampal Gyrus, posterior division Right) | -0.35 |
| atlas.SubCalC (Subcallosal Cortex) | -0.47 |
| atlas.aPaHC r (Parahippocampal Gyrus, anterior division Right) | -0.61 |
| atlas.Hippocampus l | -0.78 |
| atlas.Hippocampus r | -1.18 |
| atlas.Thalamus l | -1.64 |
| atlas.Putamen r | -1.91 |
| atlas.Amygdala r | -2.07 |
| atlas.Pallidum r | -2.13 |
| atlas.Amygdala l | -2.14 |
| atlas.Brain-Stem | -2.23 |
| atlas.Thalamus r | -2.38 |
| atlas.Putamen l | -2.42 |
| atlas.Accumbens r | -2.53 |
| atlas.Caudate l | -2.69 |
| atlas.Caudate r | -2.91 |
| atlas.Accumbens l | -3.35 |
| atlas.Pallidum l | -3.49 |
| Regions of the Conn atlas were ranked according to their averaged expression (i.e. averaged z-values) of the three marker genes GRIN1 (encoding the constitutive NMDA receptor 1 subunit), Parvalbumin and SLC17A7 (encoding the glutamatergic neural marker Vesicular glutamate transporter 1 - VGLUT1), which was obtained from the Allen Brain post mortem microarray dataset | |

| **Supplementary Table 4.** Gene expression-based ranking of atlas regions – replacement of GRIN1 by GRIN2A | |
| --- | --- |
| **Harvard Oxford Atlas Region** | **Z-value** |
| atlas.PT r (Planum Temporale Right) | 0.89 |
| atlas.PO l (Parietal Operculum Cortex Left) | 0.82 |
| atlas.ICC l (Intracalcarine Cortex Left) | 0.81 |
| atlas.OP l (Occipital Pole Left) | 0.81 |
| atlas.SPL l (Superior Parietal Lobule Left) | 0.77 |
| atlas.Cuneal l (Cuneal Cortex Left) | 0.75 |
| atlas.IFG tri r (Inferior Frontal Gyrus, pars triangularis Right) | 0.75 |
| atlas.AG r (Angular Gyrus Right) | 0.69 |
| atlas.SFG r (Superior Frontal Gyrus Right) | 0.68 |
| atlas.iLOC l (Lateral Occipital Cortex, inferior division Left) | 0.68 |
| atlas.SCC l (Supracalcarine Cortex Left) | 0.68 |
| atlas.IFG oper r (Inferior Frontal Gyrus, pars opercularis Right) | 0.66 |
| atlas.PostCG r (Postcentral Gyrus Right) | 0.65 |
| atlas.pSTG r (Superior Temporal Gyrus, posterior division Right) | 0.61 |
| atlas.toMTG r (Middle Temporal Gyrus, temporooccipital part Right) | 0.59 |
| atlas.pSTG l (Superior Temporal Gyrus, posterior division Left) | 0.58 |
| atlas.PreCG l (Precentral Gyrus Left) | 0.57 |
| atlas.sLOC l (Lateral Occipital Cortex, superior division Left) | 0.57 |
| atlas.pSMG l (Supramarginal Gyrus, posterior division Left) | 0.56 |
| atlas.iLOC r (Lateral Occipital Cortex, inferior division Right) | 0.56 |
| atlas.PreCG r (Precentral Gyrus Right) | 0.56 |
| atlas.IFG tri l (Inferior Frontal Gyrus, pars triangularis Left) | 0.55 |
| atlas.SPL r (Superior Parietal Lobule Right) | 0.55 |
| atlas.pSMG r (Supramarginal Gyrus, posterior division Right) | 0.54 |
| atlas.CO r (Central Opercular Cortex Right) | 0.54 |
| atlas.FP r (Frontal Pole Right) | 0.53 |
| atlas.OFusG l (Occipital Fusiform Gyrus Left) | 0.53 |
| atlas.IFG oper l (Inferior Frontal Gyrus, pars opercularis Left) | 0.53 |
| atlas.PostCG l (Postcentral Gyrus Left) | 0.53 |
| atlas.MidFG l (Middle Frontal Gyrus Left) | 0.52 |
| atlas.MidFG r (Middle Frontal Gyrus Right) | 0.51 |
| atlas.LG l (Lingual Gyrus Left) | 0.51 |
| atlas.SFG l (Superior Frontal Gyrus Left) | 0.51 |
| atlas.sLOC r (Lateral Occipital Cortex, superior division Right) | 0.50 |
| atlas.SMA L(Juxtapositional Lobule Cortex -formerly Supplementary Motor Cortex- Left) | 0.48 |
| atlas.Precuneous (Precuneous Cortex) | 0.48 |
| atlas.toMTG l (Middle Temporal Gyrus, temporooccipital part Left) | 0.48 |
| atlas.FP l (Frontal Pole Left) | 0.47 |
| atlas.ICC r (Intracalcarine Cortex Right) | 0.47 |
| atlas.FOrb r (Frontal Orbital Cortex Right) | 0.47 |
| atlas.TOFusC r (Temporal Occipital Fusiform Cortex Right) | 0.46 |
| atlas.Cuneal r (Cuneal Cortex Right) | 0.46 |
| atlas.toITG l (Inferior Temporal Gyrus, temporooccipital part Left) | 0.44 |
| atlas.CO l (Central Opercular Cortex Left) | 0.43 |
| atlas.toITG r (Inferior Temporal Gyrus, temporooccipital part Right) | 0.43 |
| atlas.pITG r (Inferior Temporal Gyrus, posterior division Right) | 0.42 |
| atlas.pMTG r (Middle Temporal Gyrus, posterior division Right) | 0.42 |
| atlas.PaCiG r (Paracingulate Gyrus Right) | 0.42 |
| atlas.PC (Cingulate Gyrus, posterior division) | 0.41 |
| atlas.LG r (Lingual Gyrus Right) | 0.41 |
| atlas.aMTG l (Middle Temporal Gyrus, anterior division Left) | 0.40 |
| atlas.pMTG l (Middle Temporal Gyrus, posterior division Left) | 0.39 |
| atlas.HG r (Heschls Gyrus Right) | 0.36 |
| atlas.HG l (Heschls Gyrus Left) | 0.35 |
| atlas.OP r (Occipital Pole Right) | 0.35 |
| atlas.PaCiG l (Paracingulate Gyrus Left) | 0.35 |
| atlas.AG l (Angular Gyrus Left) | 0.35 |
| atlas.aSTG r (Superior Temporal Gyrus, anterior division Right) | 0.33 |
| atlas.FO l (Frontal Operculum Cortex Left) | 0.30 |
| atlas.SCC r (Supracalcarine Cortex Right) | 0.30 |
| atlas.aSTG l (Superior Temporal Gyrus, anterior division Left) | 0.30 |
| atlas.aSMG l (Supramarginal Gyrus, anterior division Left) | 0.29 |
| atlas.MedFC (Frontal Medial Cortex) | 0.27 |
| atlas.SMA r (Juxtapositional Lobule Cortex -formerly Supplementary Motor Cortex- Right) | 0.26 |
| atlas.pITG l (Inferior Temporal Gyrus, posterior division Left) | 0.24 |
| atlas.PP r (Planum Polare Right) | 0.24 |
| atlas.TOFusC l (Temporal Occipital Fusiform Cortex Left) | 0.23 |
| atlas.aMTG r (Middle Temporal Gyrus, anterior division Right) | 0.23 |
| atlas.OFusG r (Occipital Fusiform Gyrus Right) | 0.22 |
| atlas.PO r (Parietal Operculum Cortex Right) | 0.21 |
| atlas.aITG l (Inferior Temporal Gyrus, anterior division Left) | 0.21 |
| atlas.FOrb l (Frontal Orbital Cortex Left) | 0.19 |
| atlas.PT l (Planum Temporale Left) | 0.19 |
| atlas.pTFusC r (Temporal Fusiform Cortex, posterior division Right) | 0.16 |
| atlas.FO r (Frontal Operculum Cortex Right) | 0.13 |
| atlas.aSMG r (Supramarginal Gyrus, anterior division Right) | 0.12 |
| atlas.pTFusC l (Temporal Fusiform Cortex, posterior division Left) | 0.11 |
| atlas.aITG r (Inferior Temporal Gyrus, anterior division Right) | 0.07 |
| atlas.TP l (Temporal Pole Left) | 0.02 |
| atlas.pPaHC r (Parahippocampal Gyrus, posterior division Right) | 0.00 |
| atlas.PP l (Planum Polare Left) | -0.03 |
| atlas.TP r (Temporal Pole Right) | -0.06 |
| atlas.pPaHC l (Parahippocampal Gyrus, posterior division Left) | -0.09 |
| atlas.IC r (Insular Cortex Right) | -0.16 |
| atlas.aTFusC l (Temporal Fusiform Cortex, anterior division Left) | -0.16 |
| atlas.IC l (Insular Cortex Left) | -0.22 |
| atlas.aTFusC r (Temporal Fusiform Cortex, anterior division Right) | -0.24 |
| atlas.AC (Cingulate Gyrus, anterior division) | -0.29 |
| atlas.aPaHC l (Parahippocampal Gyrus, anterior division Left) | -0.39 |
| atlas.Hippocampus l | -0.49 |
| atlas.aPaHC r (Parahippocampal Gyrus, anterior division Right) | -0.55 |
| atlas.SubCalC (Subcallosal Cortex) | -0.57 |
| atlas.Hippocampus r | -0.73 |
| atlas.Amygdala r | -1.12 |
| atlas.Amygdala l | -1.60 |
| atlas.Thalamus l | -1.66 |
| atlas.Pallidum r | -1.87 |
| atlas.Thalamus r | -2.08 |
| atlas.Putamen r | -2.20 |
| atlas.Brain-Stem | -2.29 |
| atlas.Pallidum l | -2.37 |
| atlas.Putamen l | -2.44 |
| atlas.Caudate r | -3.21 |
| atlas.Caudate l | -3.25 |
| atlas.Accumbens r | -3.30 |
| atlas.Accumbens l | -4.08 |
| Regions of the Harvard Oxford atlas were ranked according to their averaged expression (i.e. averaged z-values) of the three marker genes GRIN2A (encoding the NMDA receptor 2A subunit), Parvalbumin and SLC17A7 (encoding the glutamatergic neural marker Vesicular glutamate transporter 1 - VGLUT1), which was obtained from the Allen Brain post mortem microarray dataset | |

| **Supplementary Table 5** – Auditory-sensory-motor (ASM) regions used for the analysis of functional network connectivity with the striato-pallido-thalamic set of regions | | | | | |
| --- | --- | --- | --- | --- | --- |
| Harvard Oxford atlas region | ASM definition  from Yeo et al.17 | Definition based on meta-analysis18 | MNI coordinates from meta-analysis | | |
|  |  |  | x | y | z |
| Heschl's Gyrus Left (HG l) | yes | yes | -46 | –24 | 10 |
| Heschl's Gyrus Right (HG r) | yes | contralateral |  |  |  |
| Planum Temporale Left (PT l) | yes | no |  |  |  |
| Planum Temporale Right (PT r) | yes | no |  |  |  |
| Insular Cortex Left (IC l) | yes | contralateral |  |  |  |
| Insular Cortex Right (IC r) | yes | yes | 40 | –16 | 12 |
|  |  |  | 36 | –8 | 8 |
| Precentral Gyrus Left (PreCG l) | yes | contralateral |  |  |  |
| Precentral Gyrus Right (PreCG r) | yes | yes | 4 | –28 | 64 |
| Postcentral Gyrus Left (PostCG l) | yes | yes | -42 | –26 | 52 |
| Postcentral Gyrus Right (PostCG r) | yes | yes | 46 | –10 | 28 |
|  |  |  | 48 | –20 | 50: |
| Superior Temporal Gyrus, posterior division Left (pSTG l) | no | contralateral |  |  |  |
| Superior Temporal Gyrus, posterior division Right (pSTG r) | no | yes | 60 | –14 | -4 |
|  |  |  | 52 | –10 | -10 |
| Middle Temporal Gyrus, anterior division Left (aMTG l) | no | yes | -58 | –12 | –8 |
| Middle Temporal Gyrus, anterior division Right (aMTG r) | no | contralateral |  |  |  |
| Lateral Occipital Cortex, inferior division Left (iLOC l) | no | yes | -46 | –70 | 2 |
| Lateral Occipital Cortex, inferior division Right (iLOC r) | no | yes | 48 | –64 | -4 |
|  | | | | | |

| **Supplementary Table 6** – Connectivity changes of left cuneus | | | | | | |
| --- | --- | --- | --- | --- | --- | --- |
| Effect of GRIN2A variant (number of C-alleles) | | | | | | |
| Seed | Cluster | MNI-coordinates | | | k | Size p-FDR |
|  |  | x | y | z |  |  |
| Cuneal l | Caudate l | -08 | +14 | +06 | 135 | 0.020 |
|  | sLOC l; AG l | -46 | -56 | +52 | 417 | < 0.001 |
|  | | | | | | |
| Ketamine > midazolam | | | | | | |
|  | | | | | | |
| Region 1 | Region 2 | Statistic | | | p-uncorrected | p-FDR |
| Cuneal l | Caudate l | T(27) = 2.17 | | | 0.0195 | 0.039 |
|  | sLOC l | T(27) = 0.76 | | | 0.7731 | 0.773 |
|  | | | | | | |
| Schizophrenia > healthy control subjects | | | | | | |
|  | | | | | | |
| Region 1 | Region 2 | Statistic | | | p-uncorrected | p-FDR |
| Cuneal l - | Caudate l | T(284) = 1.54 | | | 0.062 | 0.124 |
|  | sLOC l | T(284) = 0.01 | | | 0.497 | 0.504 |
|  | Caudate r | T(284) = 2.37 | | | 0.009 | 0.037 |
|  | sLOC r | T(284) = -1.20 | | | 0.116 | 0.155 |
| Significant clusters implicated by the genetic variant were obtained from a seed to voxel analysis of the left cuneus and re-assessed in the pharmaco-fMRI and the combined schizophrenia / healthy control dataset applying ROI-to-ROI analyses (compare Figure 3). FDR = False Discovery Rate. sLOC = superior lateral occipital cortex. AG = angular gyrus; l = left. r = right. | | | | | | |

| **Supplementary Table 7** – Multivariate analysis of connections between a set of auditory-sensory-motor and striato-pallido-thalamic regions (functional network connectivity analysis) | | | |
| --- | --- | --- | --- |
| **Effect of GRIN2A variant (Nr of C-alleles)** | | | |
| **Connectivity** | | **Statistic** | **p-value** |
| Auditory-sensory-motor ROIs | Striato-pallido-thalamic ROIs | F(4,139) = 2.44 | < 0.05 |
|  | | | |
| **Region 1** | **Region 2** | **Statistic** | **p-uncorrected** |
| HG r | Caudate l | T(142) = 2.91 | 0.004 |
| pSTG r | Pallidum r | T(142) = 2.77 | 0.006 |
| HG r | Caudate r | T(142) = 2.71 | 0.008 |
| HG l | Caudate l | T(142) = 2.64 | 0.009 |
| aMTG l | Thalamus r | T(142) = 2.11 | 0.037 |
| IC r | Caudate l | T(142) = 1.99 | 0.049 |
| **ketamine > midazolam** | | | |
| **Connectivity** | | **Statistic** | **p-value** |
| Auditory-sensory-motor ROIs | Striato-pallido-thalamic ROIs | F(2,26) = 24.01 | < 0.001 |
|  | | | |
| **Region 1** | **Region 2** | **Statistic** | **p-uncorrected** |
| PostCG l | Thalamus r | T(27) = 6.73 | < 0.001 |
| PostCG l | Thalamus l | T(27) = 5.88 | < 0.001 |
| PostCG r | Thalamus r | T(27) = 5.81 | < 0.001 |
| PostCG r | Thalamus l | T(27) = 5.61 | < 0.001 |
| PreCG l | Caudate l | T(27) = 5.35 | < 0.001 |
| HG r | Caudate r | T(27) = 5.25 | < 0.001 |
| PreCG r | Thalamus r | T(27) = 4.92 | < 0.001 |
| PreCG r | Thalamus l | T(27) = 4.86 | < 0.001 |
| PreCG r | Caudate l | T(27) = 4.51 | < 0.001 |
| PreCG l | Thalamus r | T(27) = 4.39 | < 0.001 |
| aMTG l | Thalamus l | T(27) = 4.29 | < 0.001 |
| PT r | Caudate r | T(27) = 4.21 | < 0.001 |
| PostCG r | Caudate l | T(27) = 4.20 | < 0.001 |
| aMTG r | Thalamus l | T(27) = 4.15 | < 0.001 |
| pSTG r | Thalamus r | T(27) = 4.10 | < 0.001 |
| pSTG r | Thalamus l | T(27) = 4.09 | < 0.001 |
| PostCG r | Pallidum r | T(27) = 4.02 | < 0.001 |
| PostCG r | Caudate l | T(27) = 4.02 | < 0.001 |
| PreCG l | Caudate l | T(27) = 3.98 | < 0.001 |
| PreCG l | Thalamus l | T(27) = 3.84 | < 0.001 |
| PreCG l | Pallidum l | T(27) = 3.72 | < 0.001 |
| PostCG l | Pallidum r | T(27) = 3.71 | < 0.001 |
| PT l | Thalamus l | T(27) = 3.91 | < 0.001 |
| PT l | Caudate r | T(27) = 3.81 | < 0.001 |
| PT l | Caudate l | T(27) = 3.72 | < 0.001 |
| HG r | Caudate l | T(27) = 3.99 | < 0.001 |
| aMTG r | Caudate l | T(27) = 3.98 | < 0.001 |
| iLOC r | Pallidum r | T(27) = 3.86 | < 0.001 |
| PT r | Caudate l | T(27) = 3.72 | < 0.001 |
| PreCG r | Caudate r | T(27) = 3.69 | < 0.001 |
| aMTG r | Thalamus r | T(27) = 3.50 | 0.002 |
| aMTG l | Thalamus r | T(27) = 3.50 | 0.002 |
| PreCG r | Pallidum l | T(27) = 3.41 | 0.002 |
| PT r | Thalamus l | T(27) = 3.35 | 0.002 |
| HG l | Caudate r | T(27) = 3.37 | 0.002 |
| PT l | Thalamus r | T(27) = 3.29 | 0.003 |
| PostCG r | Pallidum l | T(27) = 3.24 | 0.003 |
| PT r | Thalamus r | T(27) = 3.14 | 0.004 |
| PreCG l | Pallidum r | T(27) = 3.08 | 0.004 |
| pSTG l | Thalamus l | T(27) = 3.07 | 0.005 |
| pSTG l | Thalamus r | T(27) = 3.05 | 0.005 |
| aMTG l | Caudate l | T(27) = 3.04 | 0.005 |
| iLOC l | Pallidum r | T(27) = 2.95 | 0.006 |
| PreCG r | Pallidum r | T(27) = 2.84 | 0.008 |
| PostCG l | Pallidum l | T(27) = 2.76 | 0.010 |
| PostCG l | Caudate r | T(27) = 2.75 | 0.011 |
| pSTG r | Caudate l | T(27) = 2.71 | 0.012 |
| PostCG r | Caudate r | T(27) = 2.64 | 0.014 |
| HG r | Thalamus l | T(27) = 2.63 | 0.014 |
| pSTG l | Caudate l | T(27) = 2.55 | 0.017 |
| iLOC r | Thalamus l | T(27) = 2.45 | 0.021 |
| IC r | Thalamus r | T(27) = 2.44 | 0.022 |
| HG r | Thalamus r | T(27) = 2.42 | 0.022 |
| IC l | Thalamus r | T(27) = 2.36 | 0.026 |
| aMTG r | Caudate r | T(27) = 2.35 | 0.027 |
| PT l | Pallidum l | T(27) = 2.24 | 0.033 |
| pSTG l | Caudate r | T(27) = 2.16 | 0.040 |
| aMTG l | Caudate r | T(27) = 2.05 | < 0.050 |
| **schizophrenia > healthy controls** | | | |
| **Connectivity** | | **Statistic** | **p-value** |
| Auditory-sensory-motor ROIs | Striato-pallido-thalamic ROIs | F(4,281) = 2.89 | 0.023 |
|  | | | |
| **Region 1** | **Region 2** | **Statistic** | **p-uncorrected** |
|  | |  |  |
| iLOC r | Thalamus r | T(284) = 4.37 | < 0.001 |
| iLOC r | Thalamus l | T(284) = 3.82 | < 0.001 |
| pSTG r | Pallidum l | T(284) = 3.09 | 0.002 |
| iLOC l | Thalamus l | T(284) = 2.83 | 0.005 |
| pSTG l | Thalamus r | T(284) = 2.83 | 0.005 |
| PostCG r | Thalamus l | T(284) = 2.69 | 0.007 |
| pSTG r | Thalamus r | T(284) = 2.53 | 0.012 |
| PT l | Thalamus r | T(284) = 2.43 | 0.016 |
| PT l | Thalamus l | T(284) = 2.34 | 0.020 |
| iLOC l | Thalamus r | T(284) = 2.33 | 0.021 |
| iLOC r | Pallidum l | T(284) = 2.29 | 0.023 |
| pSTG r | Thalamus l | T(284) = 2.23 | 0.027 |
| pSTG l | Thalamus l | T(284) = 2.23 | 0.027 |
| PreCG r | Thalamus l | T(284) = 2.12 | 0.035 |
| iLOC l | Pallidum l | T(284) = 2.04 | 0.042 |
| PT r | Thalamus r | T(284) = 2.04 | 0.042 |
| HG = Heschl's gyrus; PT = planum temporale; IC = insular cortex; PreCG = precentral gyrus; PostCG = postcentral gyrus; pSTG = posterior superior temporal Gyrus; aMTG = anterior middle temporal gyrus; iLOC = inferior lateral occipital cortex; l = left; r = right; | | | |

| **Supplementary Table 8** – Functional network connectivity analysis | | | | |
| --- | --- | --- | --- | --- |
| **Effect of GRIN2A variant (Nr of C-alleles)** | | | | |
|  | | | | |
| **Network pair number** | | **Statistic** | **p-uncorrected** | **p-FDR** |
| 1/210 | | F(4,139) = 6.54 | < 0.001 | 0.016 |
|  | | | | |
| **Region 1** | **Region 2** | **Statistic** | **p-uncorrected** |  |
| aSTG r | TOFusC r | T(142) = -4.68 | < 0.001 |
| pSTG l | TOFusC r | T(142) = -4.16 | < 0.001 |
| aSTG r | TOFusC l | T(142) = -4.09 | < 0.001 |
| aSTG r | iLOC l | T(142) = -4.09 | < 0.001 |
| aSTG r | iLOC r | T(142) = -3.88 | < 0.001 |
| pSTG r | TOFusC r | T(142) = -3.04 | 0.003 |
| aSTG l | iLOC l | T(142) = -2.85 | 0.005 |
| aSTG l | iLOC r | T(142) = -2.83 | 0.005 |
| pSTG l | iLOC l | T(142) = -2.70 | 0.008 |
| aSTG l | TOFusC r | T(142) = -2.67 | 0.008 |
| pSTG r | iLOC r | T(142) = -2.63 | 0.010 |
| pSTG l | OFusG r | T(142) = -2.55 | 0.012 |
| pSTG l | TOFusC l | T(142) = -2.55 | 0.012 |
| pSTG r | iLOC l | T(142) = -2.46 | 0.015 |
| pSTG l | iLOC r | T(142) = -2.41 | 0.017 |
| aSTG r | OFusG l | T(142) = -2.30 | 0.023 |
| aSTG l | TOFusC l | T(142) = -2.23 | 0.027 |
| pSTG r | OFusG l | T(142) = -2.05 | 0.042 |
| pSTG r | TOFusC l | T(142) = -1.98 | 0.049 |
| ketamine > midazolam | | | | |
| aSTG r | TOFusC r | T(27) = -2.07 | 0.024 | 0.048 |
| pSTG l | TOFusC r | T(27) = -1.14 | 0.133 | 0.133 |
| schizophrenia > healthy controls | | | | |
| aSTG r | TOFusC r | T(284) = -2.51 | 0.0063 | 0.0063 |
| pSTG l | TOFusC r | T(284) = -3.57 | 0.0002 | 0.0004 |
| Significant connectivity changes associated with the genetic variant revealed by a multivariate whole brain ROI to ROI functional connectivity (functional network connectivity) analysis. Among the 210 between or within network connections, one between network connectivity comprising connections between auditory and visual regions remained statistically significant after FDR-correction. The strongest connectivity changes were re-assessed for the contrast ketamine > midazolam and schizophrenia > healthy controls.  aSTG = anterior superior temporal gyrus; pSTG = posterior superior temporal gyrus; iLOC = inferior lateral occipital cortex; OFusG = occipital fusiform gyrus; TOFusC = temporal occipital fusiform cortex; l = left; r = right. | | | | |

| **Supplementary Table 9** – Connectivity changes of the left posterior superior temporal gyrus | | | | | | |
| --- | --- | --- | --- | --- | --- | --- |
| Effect of GRIN2A variant (number of C-alleles) | | | | | | |
| Seed | Cluster | MNI-coordinates | | | k | Size p-FDR |
|  |  | x | y | z |  |  |
| pSTG l | TOFusC r | +32 | -40 | -18 | 227 | < 0.001 |
|  | sLOC l | -30 | -84 | +20 | 241 | < 0.001 |
|  | sLOC r | +32 | -80 | +36 | 232 | < 0.001 |
|  | toITG l | -42 | -56 | -08 | 49 | 0.017 |
|  | | | | | | |
| Ketamine > midazolam | | | | | | |
|  | | | | | | |
| Region 1 | Region 2 | Statistic | | | p-uncorrected | p-FDR |
| pSTG l | sLOC l | T(27)= -1.69 | | | 0.052 | 0.104 |
|  | sLOC r | T(27)= 0.09 | | | 0.573 | 0.573 |
|  | | | | | | |
| Schizophrenia > healthy control subjects | | | | | | |
|  | | | | | | |
| Region 1 | Region 2 | Statistic | | | p-uncorrected | p-FDR |
| pSTG l | sLOC l | T(284) = -1.66 | | | 0.049 | 0.069 |
|  | sLOC r | T(284) = -1.48 | | | 0.069 | 0.069 |
| Significant clusters implicated by the genetic variant were obtained from a seed to voxel analysis of the left posterior superior temporal gyrus and re-assessed in the pharmaco-fMRI and the combined schizophrenia / healthy control dataset applying ROI-to-ROI analyses (compare Figure 7). K represents the number of voxels in each cluster. FDR = False Discovery Rate. pSTG = posterior superior temporal gyrus; TOFusC = temporo-occipital fusiform cortex; toITG = temporo-occipital inferior temporal cortex; sLOC = superior lateral occipital cortex. L = left. R = right. | | | | | | |

| **Supplementary Table 10** – Connectivity changes of left cuneus; categorical genetic variable | | | | | | |
| --- | --- | --- | --- | --- | --- | --- |
| Effect of GRIN2A variant (presence of minor allele > absence of minor allele) | | | | | | |
| Seed | Cluster | MNI-coordinates | | | k | Size p-FDR |
|  |  | x | y | z |  |  |
| Cuneal l | Caudate l | -10 | +14 | +08 | 171 | 0.004 |
|  | sLOC l; AG l | -44 | -56 | +54 | 282 | < 0.001 |
| As an alternative to our seed to voxel analysis presented in Figure 3 and Supplementary Table 6, we also conducted an exploratory analysis treating the genotype as a categorical variable differentiating between the absence (i.e. wildtype) and presence (heterozygous or homozygous carriers) of the schizophrenia associated variant (C-allele). FDR = False Discovery Rate. sLOC = superior lateral occipital cortex.AG = angular gyrus; l = left. | | | | | | |

| **Supplementary Table 11** – Multivariate analysis of connections between a set of auditory-sensory-motor and striato-pallido-thalamic regions (functional network connectivity analysis)- categorical genetic effect | | | |
| --- | --- | --- | --- |
| **Effect of GRIN2A variant (presence of minor allele > absence of minor allele)** | | | |
| **Connectivity** | | **Statistic** | **p-value** |
| Auditory-sensory-motor ROIs | Striato-pallido-thalamic ROIs | F(4,139) = 2.51 | 0.045 |
|  | | | |
| **Region 1** | **Region 2** | **Statistic** | **p-uncorrected** |
| HG r | Caudate l | T(142) = 3.59 | <0.001 |
| HG l | Caudate l | T(142) = 3.13 | 0.002 |
| HG r | Caudate r | T(142) = 3.09 | 0.002 |
| IC r | Caudate l | T(142) = 2.79 | 0.006 |
| PT l | Caudate l | T(142) = 2.59 | 0.011 |
| pSTG r | Pallidum r | T(142) = 2.30 | 0.023 |
| IC l - | Caudate l | T(142) = 2.15 | 0.033 |
| IC r - | Caudate r | T(142) = 2.00 | 0.047 |
| PT r - | Caudate l | T(142) = 1.98 | < 0.050 |
| As an alternative to our analysis presented in Figure 4 and Supplementary Table 7, we also conducted an exploratory analysis treating the genotype as a categorical variable differentiating between the absence (i.e. wildtype) and presence (heterozygous or homozygous carriers) of the schizophrenia associated variant (C-allele). FDR = False Discovery Rate. HG = Heschl's gyrus; PT = planum temporale; IC = insular cortex; pSTG = posterior superior temporal Gyrus; l = left; r = right. | | | |

| **Supplementary Table 12** – Functional network connectivity– categorical genetic effect | | | | |
| --- | --- | --- | --- | --- |
| **Effect of GRIN2A variant (presence of minor allele > absence of minor allele)** | | | | |
|  | | | | |
| **Network pair number** | | **Statistic** | **p-uncorrected** | **p-FDR** |
| 1 / 210 | | F(4,139) = 6.92 | < 0.001 | 0.009 |
|  | | | | |
| **Region 1** | **Region 2** | **Statistic** | **p-uncorrected** |  |
| pSTG l | TOFusC r | T(142) = -4.40 | < 0.001 |
| aSTG r | TOFusC r | T(142) = -3.85 | < 0.001 |
| aSTG r | TOFusC l | T(142) = -3.47 | 0.001 |
| aSTG r | iLOC l | T(142) = -3.40 | 0.001 |
| aSTG r | iLOC r | T(142) = -3.30 | 0.001 |
| pSTG l | OFusG r | T(142) = -3.13 | 0.002 |
| aSTG l | iLOC r | T(142) = -3.10 | 0.002 |
| pSTG r | TOFusC r | T(142) = -3.05 | 0.003 |
| aSTG l | iLOC l | T(142) = -3.03 | 0.003 |
| pSTG l | iLOC l | T(142) = -2.80 | 0.006 |
| pSTG l | TOFusC l | T(142) = -2.80 | 0.006 |
| pSTG l | iLOC r | T(142) = -2.72 | 0.007 |
| aSTG l | TOFusC r | T(142) = -2.61 | 0.010 |
| pSTG r | iLOC r | T(142) = -2.40 | 0.018 |
| pSTG r | iLOC l | T(142) = -2.37 | 0.019 |
| pSTG r | TOFusC l | T(142) = -2.25 | 0.026 |
| aSTG r | OFusG l | T(142) = -2.22 | 0.028 |
| aSTG l | TOFusC l | T(142) = -2.22 | 0.028 |
| pSTG l | OFusG l | T(142) = -2.16 | 0.032 |
| aSTG l | OFusG r | T(142) = -2.07 | 0.040 |
| As an alternative to our analysis presented in Figure 5 and Supplementary Table 8, we also conducted an exploratory analysis treating the genotype as a categorical variable differentiating between the absence (i.e. wildtype) and presence (heterozygous or homozygous carriers) of the schizophrenia associated variant (C-allele). FDR = False Discovery Rate. aSTG = anterior superior temporal gyrus; pSTG = posterior superior temporal gyrus; iLOC = inferior lateral occipital cortex; OFusG = occipital fusiform gyrus; TOFusC = temporal occipital fusiform cortex; l = left; r = right. | | | | |

| **Supplementary Table 13**– Connectivity changes of left posterior superior temporal gyrus and left inferior frontal gyrus, respectively; categorical genetic variable | | | | | | |
| --- | --- | --- | --- | --- | --- | --- |
| Effect of GRIN2A variant (presence of minor allele > absence of minor allele) | | | | | | |
| Seed | Cluster | MNI-coordinates | | | k | Size p-FDR |
|  |  | x | y | z |  |  |
| pSTG l | TOFusC r | +32 | -40 | -18 | 375 | < 0.001 |
|  | sLOC l | -32 | -78 | +20 | 367 | < 0.001 |
|  | sLOC r | +34 | -78 | +20 | 276 | < 0.001 |
|  | iLOC l; toITG l | -40 | -60 | -10 | 267 | < 0.001 |
|  | iLOC r; toITG r | +46 | -64 | -12 | 205 | < 0.001 |
| IFG tri l | TP I; aSTG l | -58 | +00 | -02 | 140 | 0.018 |
| As an alternative to our seed to voxel analysis of the left posterior superior temporal gyrus (pSTG; Wernicke’s area) and the left inferior frontal gyrus, pars triangularis (IFG tri; Broca’s area) as presented in Figure 6 and Supplementary Table 9, we also conducted a corresponding exploratory analysis treating the genotype as a categorical variable differentiating between the absence (i.e. wildtype) and presence (heterozygous or homozygous carriers) of the schizophrenia associated variant (C-allele). FDR = False Discovery Rate. aSTG = anterior superior temporal gyrus; pSTG = posterior superior temporal gyrus; TOFusC = temporo-occipital fusiform cortex; sLOC = superior lateral occipital cortex; iLOC = inferior lateral occipital cortex; toITG = temporo-occipital inferior temporal cortex. l = left. R = right. | | | | | | |

**References**

1. Strik W, Wopfner A, Horn H, Koschorke P, Razavi N, Walther S *et al.* The Bern psychopathology scale for the assessment of system-specific psychotic symptoms. *Neuropsychobiology* 2010; **61**(4)**:** 197-209.

2. Strik W, Stegmayer K, Walther S, Dierks T. Systems Neuroscience of Psychosis: Mapping Schizophrenia Symptoms onto Brain Systems. *Neuropsychobiology* 2017; **75**(3)**:** 100-116.

3. Adhikari BM, Dukart J, Hipp JF, Forsyth A, McMillan R, Muthukumaraswamy SD *et al.* Effects of ketamine and midazolam on resting state connectivity and comparison with ENIGMA connectivity deficit patterns in schizophrenia. *Human brain mapping* 2020; **41**(3)**:** 767-778.

4. Forsyth A, McMillan R, Campbell D, Malpas G, Maxwell E, Sleigh J *et al.* Comparison of local spectral modulation, and temporal correlation, of simultaneously recorded EEG/fMRI signals during ketamine and midazolam sedation. *Psychopharmacology (Berl)* 2018; **235**(12)**:** 3479-3493.

5. McMillan R, Forsyth A, Campbell D, Malpas G, Maxwell E, Dukart J *et al.* Temporal dynamics of the pharmacological MRI response to subanaesthetic ketamine in healthy volunteers: A simultaneous EEG/fMRI study. *J Psychopharmacol* 2019; **33**(2)**:** 219-229.

6. Schizophrenia Working Group of the Psychiatric Genomics C, Ripke S, Neale BM, Corvin A, Walters JTR, Farh K-H *et al.* Biological insights from 108 schizophrenia-associated genetic loci. *Nature* 2014; **511:** 421.

7. Pardiñas AF, Holmans P, Pocklington AJ, Escott-Price V, Ripke S, Carrera N *et al.* Common schizophrenia alleles are enriched in mutation-intolerant genes and in regions under strong background selection. *Nature Genetics* 2018; **50**(3)**:** 381-389.

8. Consortium TSWGotPG, Ripke S, Walters JT, O’Donovan MC. Mapping genomic loci prioritises genes and implicates synaptic biology in schizophrenia. 2020**:** 2020.2009.2012.20192922.

9. Trubetskoy V, Pardiñas AF, Qi T, Panagiotaropoulou G, Awasthi S, Bigdeli TB *et al.* Mapping genomic loci implicates genes and synaptic biology in schizophrenia. *Nature* 2022; **604**(7906)**:** 502-508.

10. Leucht S, Samara M, Heres S, Davis JM. Dose Equivalents for Antipsychotic Drugs: The DDD Method. *Schizophr Bull* 2016; **42 Suppl 1**(Suppl 1)**:** S90-94.

11. Hawrylycz MJ, Lein ES, Guillozet-Bongaarts AL, Shen EH, Ng L, Miller JA *et al.* An anatomically comprehensive atlas of the adult human brain transcriptome. *Nature* 2012; **489**(7416)**:** 391-399.

12. Arnatkevic̆iūtė A, Fulcher BD, Fornito A. A practical guide to linking brain-wide gene expression and neuroimaging data. *NeuroImage* 2019; **189:** 353-367.

13. Greicius MD, Kiviniemi V, Tervonen O, Vainionpaa V, Alahuhta S, Reiss AL *et al.* Persistent default-mode network connectivity during light sedation. *Human brain mapping* 2008; **29**(7)**:** 839-847.

14. Muthukumaraswamy SD, Shaw AD, Jackson LE, Hall J, Moran R, Saxena N. Evidence that Subanesthetic Doses of Ketamine Cause Sustained Disruptions of NMDA and AMPA-Mediated Frontoparietal Connectivity in Humans. *J Neurosci* 2015; **35**(33)**:** 11694-11706.

15. Avram M, Brandl F, Bäuml J, Sorg C. Cortico-thalamic hypo- and hyperconnectivity extend consistently to basal ganglia in schizophrenia. *Neuropsychopharmacology* 2018; **43**(11)**:** 2239-2248.

16. Jafri MJ, Pearlson GD, Stevens M, Calhoun VD. A method for functional network connectivity among spatially independent resting-state components in schizophrenia. *Neuroimage* 2008; **39**(4)**:** 1666-1681.

17. Yeo BT, Krienen FM, Sepulcre J, Sabuncu MR, Lashkari D, Hollinshead M *et al.* The organization of the human cerebral cortex estimated by intrinsic functional connectivity. *J Neurophysiol* 2011; **106**(3)**:** 1125-1165.

18. Ramsay IS. An Activation Likelihood Estimate Meta-analysis of Thalamocortical Dysconnectivity in Psychosis. *Biol Psychiatry Cogn Neurosci Neuroimaging* 2019; **4**(10)**:** 859-869.

19. Sorensen TA. A method of establishing groups of equal amplitude in plant sociology based on similarity of species content and its application to analyses of the vegetation on Danish commons. *Biol Skar* 1948; **5:** 1-34.

20. Niu H-M, Yang P, Chen H-H, Hao R-H, Dong S-S, Yao S *et al.* Comprehensive functional annotation of susceptibility SNPs prioritized 10 genes for schizophrenia. *Translational Psychiatry* 2019; **9**(1)**:** 56.

21. Ng B, White CC, Klein HU, Sieberts SK, McCabe C, Patrick E *et al.* An xQTL map integrates the genetic architecture of the human brain's transcriptome and epigenome. *Nature neuroscience* 2017; **20**(10)**:** 1418-1426.

22. Collado-Torres L, Burke EE, Peterson A, Shin J, Straub RE, Rajpurohit A *et al.* Regional Heterogeneity in Gene Expression, Regulation, and Coherence in the Frontal Cortex and Hippocampus across Development and Schizophrenia. *Neuron* 2019; **103**(2)**:** 203-216.e208.

23. Dillman AA, Majounie E, Ding J, Gibbs JR, Hernandez D, Arepalli S *et al.* Transcriptomic profiling of the human brain reveals that altered synaptic gene expression is associated with chronological aging. *Scientific Reports* 2017; **7**(1)**:** 16890.

24. Siu CR, Beshara SP, Jones DG, Murphy KM. Development of Glutamatergic Proteins in Human Visual Cortex across the Lifespan. *The Journal of Neuroscience* 2017; **37**(25)**:** 6031.

25. Liu C, Cheng L, Badner JA, Zhang D, Craig DW, Redman M *et al.* Whole-genome association mapping of gene expression in the human prefrontal cortex. *Molecular psychiatry* 2010; **15**(8)**:** 779-784.

26. Stranger BE, Nica AC, Forrest MS, Dimas A, Bird CP, Beazley C *et al.* Population genomics of human gene expression. *Nature Genetics* 2007; **39**(10)**:** 1217-1224.
