# Supplementary figures and images for: Functional connectivity signatures of NMDAR dysfunction in schizophrenia—integrating findings from imaging genetics and pharmaco-fMRI
Source: Transl Psychiatry. 2023 Feb 16;13:59. doi: 10.1038/s41398-023-02344-2 (PMC9935542; doi:10.1038/s41398-023-02344-2)

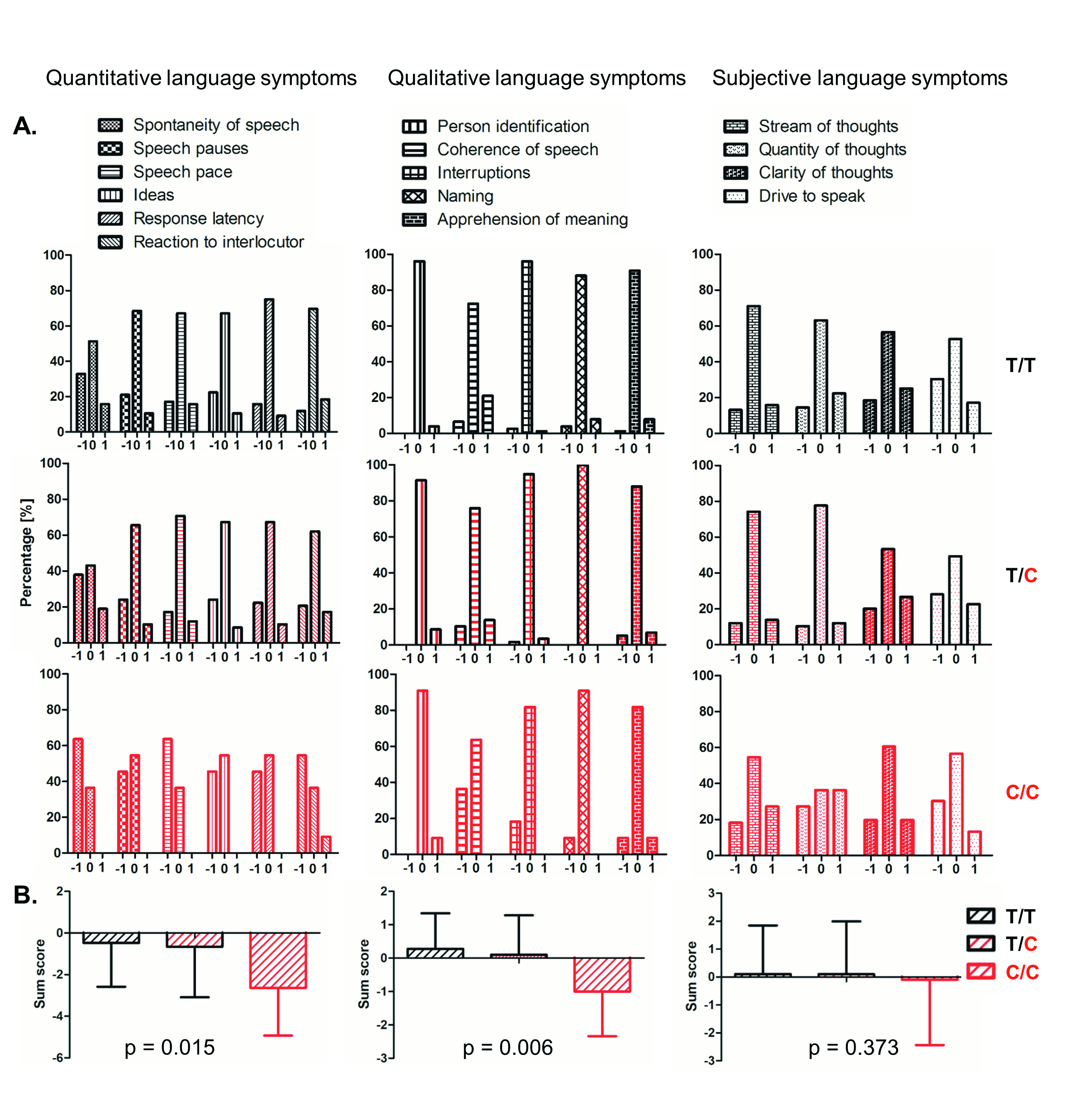

Supplement: Supplementary file 2 — Supplementary Figure 1 [file 41398_2023_2344_MOESM2_ESM.tif]

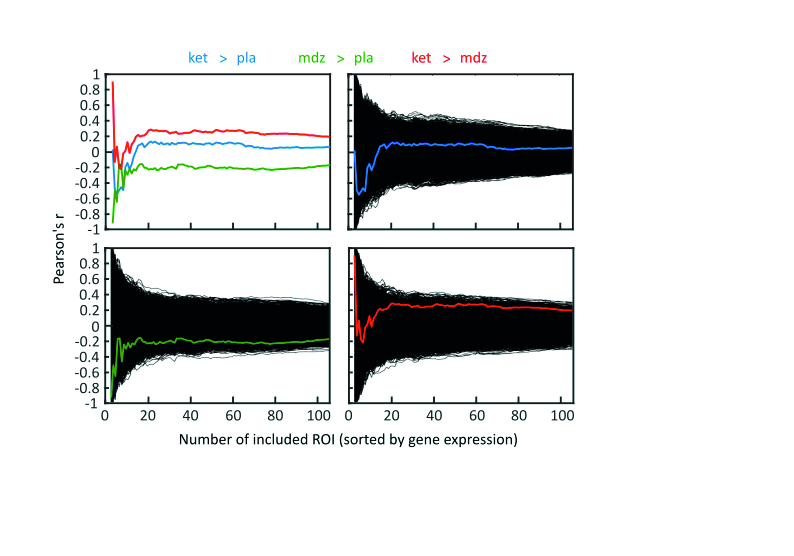

Supplement: Supplementary file 3 — Supplementary Figure 2 [file 41398_2023_2344_MOESM3_ESM.tif]

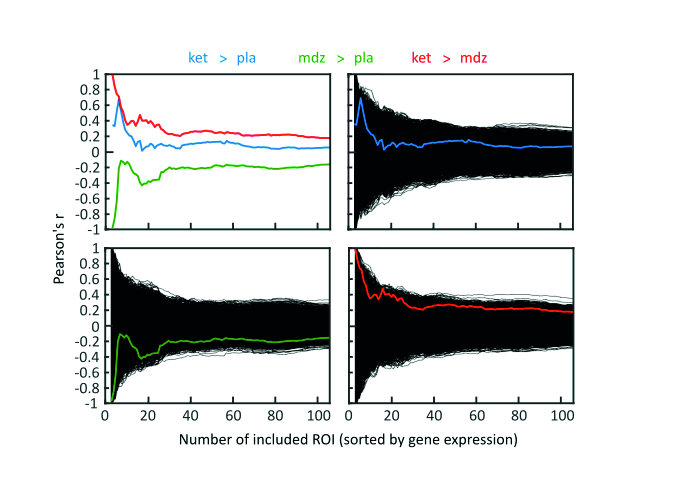

Supplement: Supplementary file 4 — Supplementary Figure 3 [file 41398_2023_2344_MOESM4_ESM.tif]

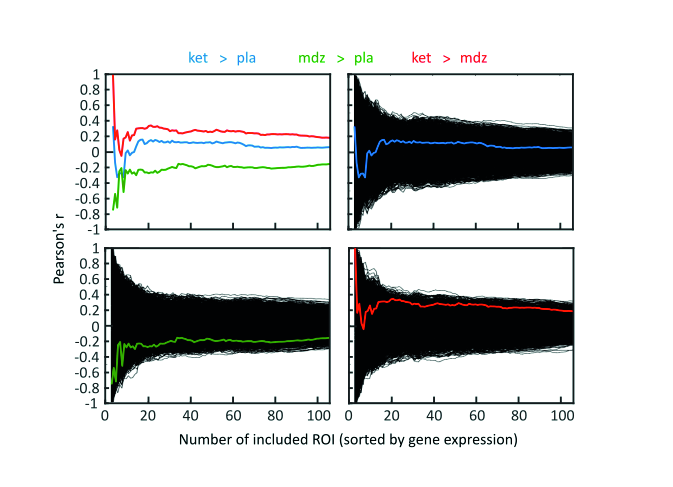

Supplement: Supplementary file 5 — Supplementary Figure 4 [file 41398_2023_2344_MOESM5_ESM.tif]
